# Gag-Pol Processing during HIV-1 Virion Maturation: A Systems Biology Approach

**DOI:** 10.1371/journal.pcbi.1003103

**Published:** 2013-06-06

**Authors:** Balázs Könnyű, S. Kashif Sadiq, Tamás Turányi, Rita Hírmondó, Barbara Müller, Hans-Georg Kräusslich, Peter V. Coveney, Viktor Müller

**Affiliations:** 1 Institute of Biology, Eötvös Loránd University, Budapest, Hungary; 2 Computational Biophysics Laboratory (GRIB-IMIM), Universitat Pompeu Fabra, Barcelona, Spain; 3 Institute of Chemistry, Eötvös Loránd University, Budapest, Hungary; 4 Institute of Enzymology, Hungarian Academy of Sciences, Budapest, Hungary; 5 Department of Infectious Diseases, Virology, University of Heidelberg, Heidelberg, Germany; 6 Centre for Computational Science, Christopher Ingold Laboratories, University College London, London, United Kingdom; 7 Research Group of Theoretical Biology and Evolutionary Ecology, Eötvös Loránd University and the Hungarian Academy of Sciences, Budapest, Hungary; University of Texas at Austin, United States of America

## Abstract

Proteolytic processing of Gag and Gag-Pol polyproteins by the viral protease (PR) is crucial for the production of infectious HIV-1, and inhibitors of the viral PR are an integral part of current antiretroviral therapy. The process has several layers of complexity (multiple cleavage sites and substrates; multiple enzyme forms; PR auto-processing), which calls for a systems level approach to identify key vulnerabilities and optimal treatment strategies. Here we present the first full reaction kinetics model of proteolytic processing by HIV-1 PR, taking into account all canonical cleavage sites within Gag and Gag-Pol, intermediate products and enzyme forms, enzyme dimerization, the initial auto-cleavage of full-length Gag-Pol as well as self-cleavage of PR. The model allows us to identify the rate limiting step of virion maturation and the parameters with the strongest effect on maturation kinetics. Using the modelling framework, we predict interactions and compensatory potential between individual cleavage rates and drugs, characterize the time course of the process, explain the steep dose response curves associated with PR inhibitors and gain new insights into drug action. While the results of the model are subject to limitations arising from the simplifying assumptions used and from the uncertainties in the parameter estimates, the developed framework provides an extendable open-access platform to incorporate new data and hypotheses in the future.

## Author Summary

Human Immunodeficiency Virus (HIV) produces its structural proteins and key enzymes in the form of polyproteins, from which the individual proteins need to be released in a complex and tightly regulated series of cleavage reactions to give rise to a morphologically mature, infectious virus particle. This process is catalyzed by a viral protease (PR), which is itself embedded in one of the polyproteins, and is one of the main targets of antiretroviral drugs. We have developed the first full reaction kinetics model that addresses the several layers of complexity (multiple cleavage sites and substrates; multiple enzyme forms; PR auto-processing) associated with the proteolytic processing of HIV polyproteins. The model allows us to identify the rate limiting step of virion maturation and the parameters with the strongest effect on maturation kinetics. We predict how changes in the individual cleavage rates and the effects of drugs might interact and possibly compensate each other, characterize the detailed time course of the process, and explain why the effectiveness of PR inhibitors rises very steeply at a critical threshold concentration of the drugs. These new insights promote our understanding of the viral life cycle and may guide the future development of antiviral drugs.

## Introduction

**Figure 1:**
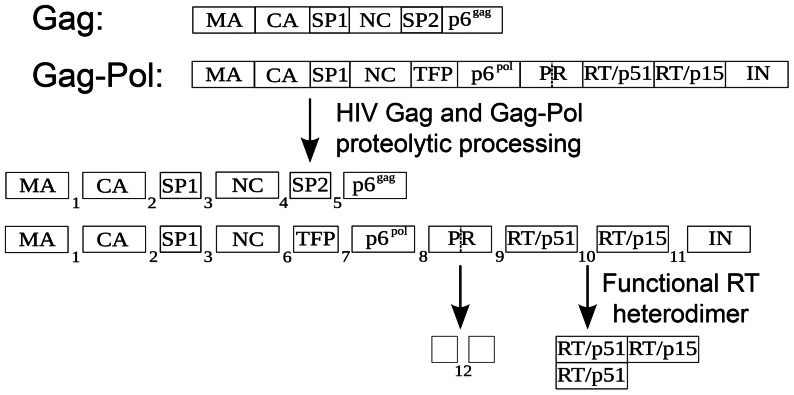
The scheme of Gag and Gag-Pol processing by HIV-1 PR. Our model includes the 11 canonical cleavage sites at which the PR cleaves the polyproteins to release the functional enzymes and structural proteins of the virus (indicated by numbers 1–11). A cleavage site within PR itself that mediates auto-inactivation of proteolytic activity is indicated by a dashed line (12). RT is active as a heterodimer of p51 and p66 (p51 and p15 still uncleaved). Abbreviations: MA – matrix, CA – capsid, SP1 – spacer peptide 1, NC – nucleocapsid, SP2 – spacer peptide 2, TFP – transframe peptide, PR – protease, RT – reverse transcriptase, IN – integrase. Boxes representing individual domains are not drawn to scale.

The morphological maturation of human immunodeficiency virus type 1 (HIV-1) depends on the proteolytic processing of the Gag and Gag-Pol polyproteins by the virus encoded PR that occurs concomitant with or shortly after virus release [1]. PR inhibitors (PIs) that interfere with this process result in the production of immature, noninfectious virus particles, and constitute an important drug class in anti-HIV-1 therapy [2], [3]. While currently approved drugs act by competitive binding to the PR active site, thereby affecting all cleavage events, individual steps of the maturation process are also potential targets for future drug development [4], [5]. The development and therapeutic application of HIV-1 maturation inhibitors requires a detailed understanding of the cleavage process, which has multiple layers of complexity. First, PR itself is embedded in the Gag-Pol polyprotein, and Gag-Pol auto-processing is required to initiate the maturation process [6]–[9]. Liberated PR molecules then catalyze further cleavage events, which might result in accelerated PR release by a positive feedback loop. Second, due to its relatively broad substrate specificity [10], HIV PR targets 11 canonical cleavage sites in the Gag and Gag-Pol polyproteins (Figure 1), generating 66 distinct molecular species (substrates, intermediates and products), and a large number of competing reactions occur simultaneously within the confined space of the virion. Cleavage at the individual sites occurs with different rates; a 400-fold difference in rate between the fastest (SP1-NC) and slowest (CA-SP1) cleavage site in Gag has been determined *in vitro* [11]. Third, several intermediates include an active protease domain, but also one or more uncleaved cleavage sites: these molecular species have dual roles as both substrates and enzymes in the reaction network. The complexity of the reaction system (number of reactions/reactants) is comparable to that of the cell cycle, which has been among the most important targets of systems modelling in biology so far [12].

Understanding and predicting the behaviour of this complex system requires systems modelling and extensive empirical data to parameterize the model. Accumulating data on the kinetic parameters of the cleavage reactions [6]–[9], [13]–[20] and on the biology of HIV-1 virion assembly and maturation [21]–[26] now allow us to tackle this problem, and we here present the first full reaction kinetics model of proteolytic processing by HIV-1 PR. We use the model to characterize the general time course of the process, to identify the parameters with the strongest effect on the maturation process, to assess interactions between the parameters and the potential of individual parameters to compensate for drug effects or changes in other parameters, and to explain the steep dose response curves associated with PIs [27]. While subject to inevitable limitations arising from the simplifying assumptions used and from uncertainty in the parameter estimates, our model results provide a level of resolution exceeding that of currently available experimental data. The time course of proteolytic processing has been characterized quantitatively for purified Gag *in vitro* [11], but was determined only qualitatively for Gag-Pol [28], and experiments are typically limited to tracking a small number of molecular species in simplified *in vitro* systems. In contrast, the model presented here tracks all intermediates and products of the complex reaction system. Furthermore, the model can be used to predict the effect of quantitatively characterized mutations or drugs alone or in combination. It can also be applied to predict the effect of potential perturbations induced by compounds in drug development. Systems modelling has been used to identify and characterize synergistic drug interactions that can enhance the effect of drugs [29], [30]. The model presented here opens the possibility to apply this approach to HIV-1 PR inhibition.

## Results

### Time course of Gag-Pol processing and virion maturation

**Figure 2:**
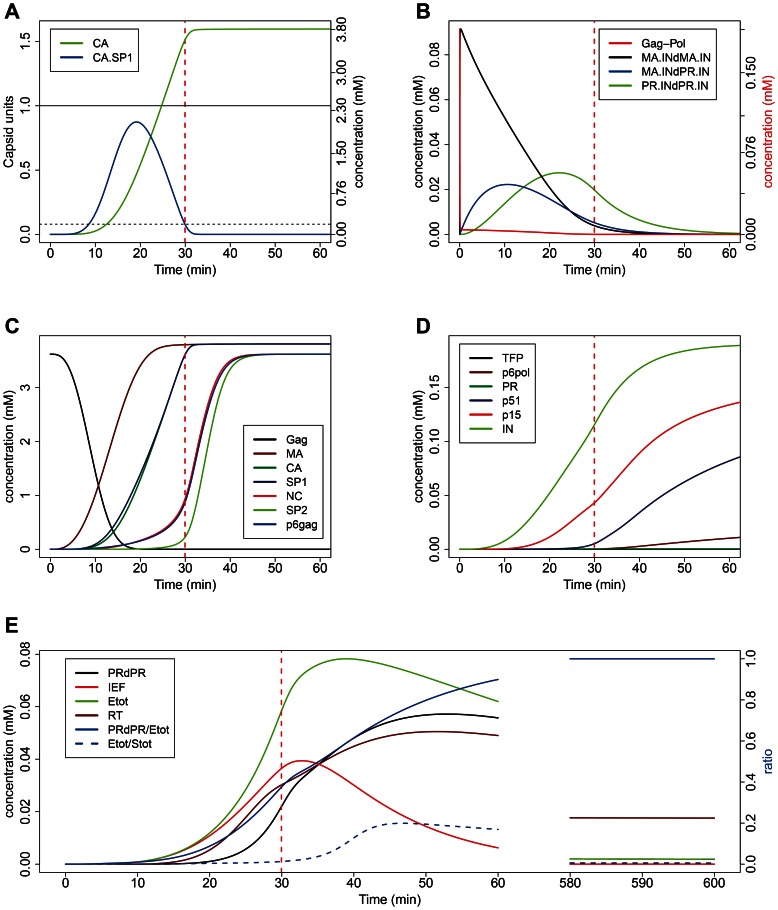
The time course of simulated Gag and Gag-Pol processing. Initial concentrations of Gag and Gag-Pol were set to reflect the quantities within a single virion; cleavage rates were parameterized according to *in vitro* estimates (Table 1). (A) Virus maturation time (VMT) as defined by the molecular species known to govern virion maturation: Morphological maturation (indicated by dashed red line in all panels) is triggered by the decay of the CA.SP1 fragment (blue line; threshold of trans-dominant inhibition of particle maturation indicated by dashed horizontal line) and is not limited by the availability of liberated CA molecules (green line; threshold of one capsid unit corresponding to 1,500 CA molecules per particle is indicated by solid horizontal line). (B) Generation of catalytically active intermediate dimeric forms containing PR. Full-length Gag-Pol (red line) dimerizes rapidly and N-terminal auto-cleavage gives rise to enzymatically active intermediate dimeric forms (black, blue and green lines). (C) Decay of Gag substrate (black line) and accumulation of final Gag cleavage products. (D) Accumulation of final Pol cleavage products. (E) Enzyme concentrations and related metrics. The ratio PRdPR/E_tot_ indicates the relative contribution of mature PR dimers to the proteolytic activity. The ratio E_tot_/S_tot_ of the total concentration of active enzyme forms and the total concentration of uncleaved cleavage sites stays below one throughout the simulated time course, which justifies the use of Michaelis-Menten kinetics. E_tot_ – total proteolytic activity; S_tot_ – all uncleaved cleavage sites; IEF – all active intermediate enzyme (PR) forms; RT: p51/p66 heterodimer. All other dimers are indicated in the form M_1_dM_2_, where M_1,2_ are the monomers.

We built a full model of PR catalyzed Gag- and Gag-Pol processing based on mass action Michaelis-Menten reaction kinetics (Materials and Methods). Based on published data on the architecture of the immature HIV-1 particle [22], we set initial concentrations to correspond to 2,280 molecules of Gag (corresponding to 3.619 mM) and 120 molecules of Gag-Pol (corresponding to 0.195 mM) within the confined space of a spherical virus particle with a radius of 63 nm [22] as the starting point for our simulations. By parameterizing all cleavage reactions according to *in vitro* empirical estimates (Table 1), our model generated a detailed predicted time course for the cleavage process (Figure 2; major intermediates are shown in Figure S1). The timing of virion maturation (virion maturation time, VMT; dashed red line in all panels of Figure 2) was estimated based on two criteria for maturation: i) the presence of a sufficient number of liberated CA molecules to form a mature conical capsid (one “capsid unit” corresponding to 1,500 CA monomers [31]), and ii) the concentration of the late processing intermediate CA.SP1 falling below a critical level. Processing at this site is required for mature capsid formation [26], [32]–[34]. A CA.SP1 concentration below 5% of the total initial Gag content is needed to fully alleviate the trans-dominant inhibition effect of this fragment on HIV-1 infectivity [35], and we used this threshold as a criterion for attaining VMT. The time needed for the assembly of the mature cone shaped capsid was not considered in our definition of the VMT, which implies that our estimates for VMT can be regarded as lower bounds; however, assembly is likely to be fast compared with the preceding steps of proteolytic processing (see Discussion). Using the default parameters, our model predicted morphological maturation to occur ∼30 min after the start of the process, which is thought to be initiated at the formation of the virion. While there are no reliable data on the timing of virion maturation *in vivo*, the fact that morphological maturation intermediates have not been detected by electron microscopy indicates that the process is comparatively fast. Our result is roughly consistent with the current assumption that maturation occurs during or shortly after budding [36], taken together with fluorescence imaging results indicating that most HIV-1 virions are released from the cell within 30 min after formation [37], apparently with a ∼15 min delay after the assembly of the Gag shell beneath the cell membrane [23].

**Table 1:** Parameters used for the simulation of the model^a^.

Parameter	pH	Value	Unit	References
k_cat_ MA/CA	5.6	6.8	s^−1^	[17]
K_M_ MA/CA	5.6	0.15	mM	[17]
k_cat_ CA/SP1	5.6	0.09	s^−1^	[13]
K_M_ CA/SP1	5.6	0.01	mM	[13]
k_cat_ SP1/NC	5.6	3.7	s^−1^	[13]
K_M_ SP1/NC	5.6	0.05	mM	[13]
k_cat_ NC/SP2	5.6	0.15	s^−1^	[16]
K_M_ NC/SP2	5.6	0.17	mM	[16]
k_cat_ SP2/p6^gag^	5.6	0.98	s^−1^	[16]
K_M_ SP2/p6^gag^	5.6	1.2	mM	[16]
k_cat_ NC/TFP	5.0	1.9×10^−5^	s^−1^	[18] b
K_M_ NC/TFP	5.0	0.05	mM	[18] b
k_cat_ TFP/p6^pol^	5.0	2.0×10^−4^	s^−1^	[18] b
K_M_ TFP/p6^pol^	5.0	0.05	mM	[18] b
k_cat_ p6^pol^/PR	5.6	0.06	s^−1^	[13]
K_M_ p6^pol^/PR	5.6	8.7×10^−3^	mM	[13]
k_cat_ PR/p51	5.6	1.5	s^−1^	[13]
K_M_ PR/p51	5.6	0.07	mM	[13]
k_cat_ p51/p15	5.6	0.4	s^−1^	[13]
K_M_ p51/p15	5.6	0.04	mM	[13]
k_cat_ p15/IN	5.6	1.2	s^−1^	[13]
K_M_ p15/IN	5.6	6.0×10^−3^	mM	[13]
k_cat_ in PR	5.5	2.85	s^−1^	[15] c
K_M_ in PR	5.5	0.215	mM	[15] c
k_cat_ auto-cleavage	5.0/7.0	10^−3^	s^−1^	[7], [8] d
k_ass_ PRdPR	6.0	1.02×10^3^	mM^−1^ s^−1^	[19]
k_diss_ PRdPR	6.0	2.15×10^−3^	s^−1^	[19]
k_ass_ IEF^e^		1.02×10^2^	mM^−1^ s^−1^	see below^f^
k_diss_ IEF^e^		2.15×10^−3^	s^−1^	see below^f^
k_ass_ Gag-Pol^g^		1.02×10^1^	mM^−1^ s^−1^	see below^f^
k_diss_ Gag-Pol^g^		2.15×10^−3^	s^−1^	see below^f^
k_ass_ RT		14	mM^−1^ s^−1^	[87]
k_diss_ RT		4.5×10^−3^	s^−1^	[87]
k_ass_ Darunavir		2.2×10^3^	mM^−1^ s^−1^	[43]
k_diss_ Darunavir		7.8×10^−7^	s^−1^	[43]
Initial concentration of Gag		3.619	mM	Based on [22]
Initial concentration of Gag-Pol		0.1905	mM	Based on [22]

a Many parameters are very sensitive to pH (for example Gag-Pol auto-cleavage [8], [9], PR dimerization [14] and also the rates of heteromolecular cleavage). The pH within virions is unclear; however, it may be assumed to be close to the optimum for PR activity (pH∼5.5), we have therefore selected parameter estimates obtained at this pH wherever available.

Initial concentrations were calculated based on data from [22] as follows. Radius of the virus: r = 63 nm = 6.3×10^−7^ dm; number of Gag molecules: 2280; number of Gag-Pol molecules: 120 (total Gag content: 2400). The volume of a virion V = (4π(r^3^))/3≈1.05×10^−18^ dm^3^. Considering that 1 mol consists of 6×10^23^ molecules, (2280/(6×10^23^) = 3.8×10^−21^ mol and 120/(6×10^23^) = 2×10^−22^ mol, then 3.8×10^−21^/1.05×10^−18^≈3.619×10^−3^ mol/dm^3^ and 2×10^−22^/1.05×10^−18^ = 1.9047×10^−4^ mol/dm^3^.

b No direct estimates were available for these two cleavage sites. We used the median of the *K_M_* values estimated for the other canonical cleavage sites, and estimated *k_cat_* from data presented in [18] (see supplementary Text S1).

c Reflecting the fastest of three internal cleavage sites reported in [15].

d The mean of the two reported values (1.3 and 0.7×10^−3^) was used.

e Intermediate forms (dimers) with liberated N-termini but C-terminal flanking fragments still uncleaved in at least one of the monomers.

f No empirical estimates were available for full length Gag-Pol and intermediate enzyme forms. Presumed decreased efficiency was implemented by decreasing the association rate with one and two orders of magnitude, respectively, compared with the mature PR.

g In addition to full-length Gag-Pol dimer, this category included the first intermediate product of auto-cleavage: MA.INTdPR.INT.

Abbreviations in the table: k_cat_ – catalytic rate constant; K_M_ – Michaelis constant; k_ass_ – association rate constant; k_diss_ – dissociation rate constant.

Total proteolytic activity (Figure 2E, green line) peaks around 39 min, and declines afterwards due to the internal cleavage of PR monomers. Remarkably, the combined catalytic activity of all intermediate enzyme forms exceeds that of the fully cleaved PR homodimer until ∼35 min after the start of the process (Figure 2E, blue line indicates the relative contribution of mature PR dimers to the proteolytic activity). Up to the time of VMT (dashed red line), intermediate enzyme forms are predicted to have catalyzed ∼80% of all cleavage reactions. Functional p66/p51 heterodimers of reverse transcriptase (RT) also decline after a peak due to the cleavage of the p66 subunit into p51 and p15 fragments; however, this decay is arrested as PR activity is lost (this might occur even faster *in vivo*: see Discussion). Finally, we have verified that the total concentration of uncleaved cleavage sites greatly exceeds the total concentration of active enzyme forms throughout the simulated cleavage process (Figure 2E), which justifies the use of Michaelis-Menten kinetics (assuming quasi steady state for the enzyme–substrate complexes).

**Figure 3:**
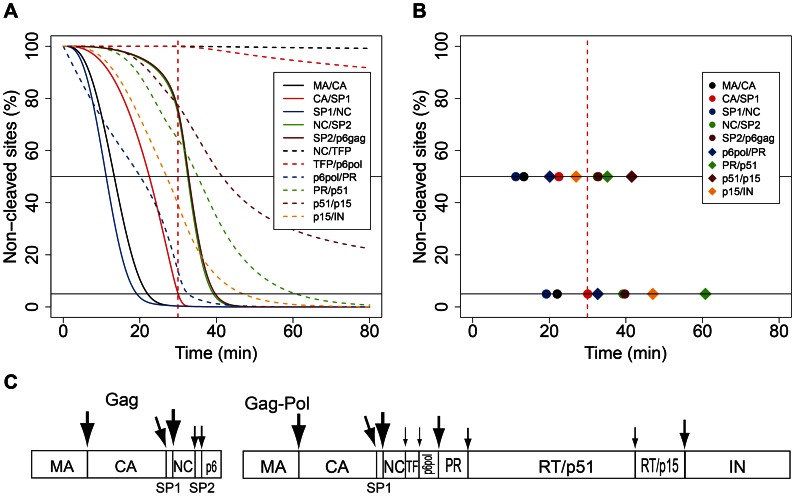
The processing of canonical cleavage sites in the simulations of Gag and Gag-Pol processing. (A) The fraction of a given cleavage site that is yet uncleaved (the total concentration of all molecular species that contain the uncleaved site, divided by the initial concentration of Gag or Gag-Pol, respectively). (B) The order of cleavage defined by the time points when a fixed threshold of processing (50% and 95%), indicated by horizontal lines, has been reached. (C) Schematic representation of the proposed order of processing events in HIV-1 Gag and Gag-Pol, respectively. The size of the arrowheads indicates different rates of cleavage, with large arrowheads representing faster cleavage. The order of processing for Gag is based on the times needed to attain 50% processing in the model, as in (B). Note that the sizes of arrowheads are not drawn to scale.

We also plot the time course of the overall processing of individual cleavage sites in Figure 3A: the figure shows what fraction of a given cleavage site is yet uncleaved (the total concentration of all molecular species that contain the uncleaved site, divided by the initial concentration). The order of cleavage can be defined for fixed thresholds of processing: Figure 3B depicts the order obtained for 50% and 95% processing; Figure 3C presents a schematic representation of the order of cleavage events based on 50% processivity. The order of events in our simulations is roughly consistent with the order of events observed *in vitro* [11], [38], with two exceptions: the removal of the spacer peptide from CA and the cleavage at the N-terminus of PR (p6^pol^/PR) occur much faster in the simulations than *in vitro*. These discrepancies arise from the relatively faster rates of cleavage observed during the processing of oligopeptides, which were used to parameterize the model. However, slowing down the processing of the CA/SP1 site to reproduce the results of *in vitro* processing of full-length Gag (as in [39]) results in VMT>2 hours (see Discussion); we therefore used the parameter set derived from oligopeptide cleavage (Table 1) in the subsequent analyses.

We thus conclude that our model is able to capture most known characteristics of the cleavage process, and proceed to analyze further properties of the system, for which little or no empirical data exist yet.

### Rates of Gag-Pol auto-cleavage and CA/SP1 cleavage set the time scale for virion maturation

**Figure 4:**
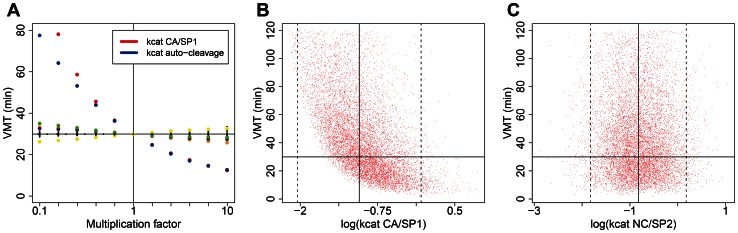
The effect of parameter variation on the virion maturation time (VMT). (A) The effect of varying each parameter individually along a geometric series between 0.1 to 10 times its default value, while fixing all other parameters at their default values. Color symbols depict VMT as a function of the parameters with the strongest effects (*k_cat_* of CA/SP1: red; rate constant of Gag-Pol auto-cleavage: blue; *K_M_* of NC/SP2: green; *K_M_* (yellow) and *k_cat_* (brown) of p6^pol^/PR; *k_d_* of Gag-Pol: purple); the effect of variation in all other parameters is illustrated by black symbols, which are overlaid on the horizontal line positioned at default VMT, indicating no discernible effect. (B–C) Multivariate exploration of the parameter space. All parameters were drawn randomly from lognormal distributions parameterized such that 95% of the values fell in the range of 0.1 to 10 times the default value of the parameters, or 0.01 to 100 times the default for the parameters with no direct empirical estimates. Default parameter values are indicated by solid vertical lines, the limits of the 95% range by dashed vertical lines. The catalytic rate constant of CA/SP1 cleavage (B) had a strong impact on VMT; the rate constant of Gag-Pol auto-cleavage had very similar effect. None of the other parameters had any discernible effect on VMT: (C) shows a representative example with VMT plotted against the catalytic rate constant of NC/SP2 cleavage. Results from 10,000 simulations are shown; default VMT is indicated by solid horizontal line; parameters were plotted on a log scale in all three panels, with a dimension of s^−1^ in B–C.

We next investigated the sensitivity of the maturation time to the parameters of the model. These analyses provide insight into the sensitivity of the results to the uncertainty of the parameters, and also predict the response of the system to possible interventions that affect individual steps of the process. We first varied one parameter at a time (see Table 1 for the list of all 33 parameters) in the range of 0.1 to 10 times its default value, while fixing all other parameters at their default values (Figure 4A). Within the studied range, varying most parameters had hardly any effect on the virus maturation time, with the exception of two critical parameters, which emerged as dominant factors: the rate constant of auto-cleavage by the full length Gag-Pol dimers, and the catalytic rate constant of heteromolecular cleavage at the CA/SP1 cleavage site. The dependence of VMT on both dominant parameters was very similar: at the lower (slower) end of the studied range, VMT is very sensitive to small changes in these parameters, while at the higher (faster) end, further increase in either rate constant yields diminishing reductions in VMT. We also tested the effect of initial Gag content of the virus. Since HIV-1 particles are not homogeneous, but have been shown to vary with respect to diameter [40] and completeness of the spherical Gag shell [22], [25], this parameter will vary among individual virions [41]. Varying initial conditions from 1,600 to 3,500 molecules of total Gag content (while keeping the Gag∶Pol ratio of 20∶1 constant) had negligible effect on the time course of virion maturation (variation in VMT was ≤1 second).

We next performed a multivariate exploration of the parameter space. Parameters were drawn randomly from lognormal distributions parameterized such that 95% of the values fell in the range of 0.1 to 10 times the default value of the parameters; for the few parameters with no direct empirical estimates (the association and dissociation rate constants of full-length Gag-Pol and partially cleaved PR enzyme forms, and the *K_M_* values for the NC/TFP and TFP/p6^pol^ cleavage sites), we allowed a range with plus/minus two orders of magnitude around the default value. We performed 10,000 simulation runs with independently generated random parameter sets, of which 8,937 achieved virion maturation by 120 min. Median VMT (when censoring uncompleted runs at VMT = 121 min) was 38.5 min (IQR: 22.4–66.6 min); the distribution of VMT was non-normal (Kolmogorov-Smirnov test, p<10^−10^; Figure S2). Maturation was triggered by the loss of CA.SP1 inhibition in 7,141 (80%) of the cases where maturation occurred, and we verified that the criterion for the Michaelis-Menten approximation (S_tot_>E_tot_) was fulfilled for nearly all (>99%) parameter sets. The dominance of the catalytic rate constants of initial auto-cleavage and CA/SP1 cleavage was confirmed in this analysis. Of the 33 parameters, only four had a significant effect on VMT (Spearman rank correlation test; p<0.0015 after Bonferroni correction): this included both dominant rate constants, which also displayed considerable correlation strength (Spearman's Rho of −0.58 and −0.45 for the CA/SP1 catalytic rate constant and for the rate constant of Gag-Pol auto-cleavage, respectively). The catalytic rate constant of the NC/SP2 cleavage and the association rate constant of active (N-terminally free) partially cleaved PR forms also affected the VMT according to this analysis, but displayed only very weak correlation (Spearman's Rho around −0.03). Only the two dominant rate constants had discernible impact on the distribution of plotted VMT values (Figures 4B and C show the influence of a dominant rate constant and of a representative “neutral” rate constant, respectively).

We thus conclude that the time-limiting steps in virion maturation (with current maturation criteria) are the initial auto-cleavage of full-length Gag-Pol and the processing of the CA/SP1 cleavage site.

### Compensation and interactions between the dominant rate constants

**Figure 5:**
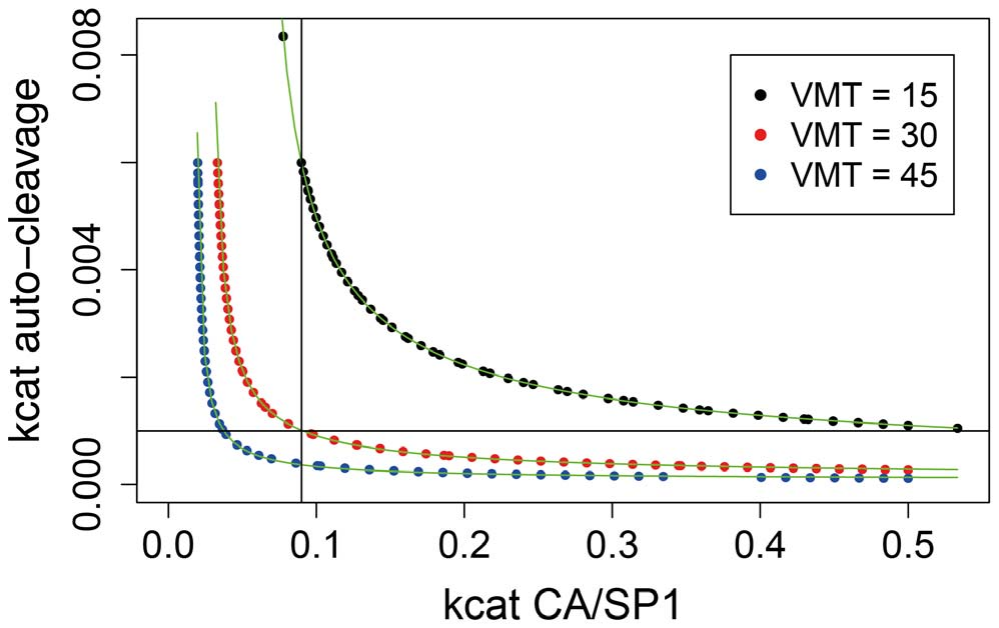
Isoclines of virion maturation time (VMT). The isoclines are drawn in the plane of the two rate parameters with the strongest effect on VMT. Generic hyperbola-like functions of the form y = a+b/(x−c)^d^ could be fitted to the data. For VMT = 15 min: a = 10^−4^, b = 5×10^−4^, c = 0.058, d = 0.69; for VMT = 30 min: a = 10^−4^, b = 10^−4^, c = 0.027, d = 0.79; for VMT = 45 min: a = 9×10^−5^, b = 2×10^−5^, c = 0.017, d = 0.93; dimensions for parameters a–c and for both axes of the figure are s^−1^. The perpendicular lines indicate the default values of both parameters.

Given the comparable magnitude of the impact of both dominant parameters on VMT, any effect (mutation or drug) involving one of the rate constants might be compensated by a change in the other. We investigated the potential for such compensation and for interactions (synergy [29], [30] or antagonism) between the rate constants. Figure 5 shows isoclines of VMT (isoboles [30]) with the rate constant of Gag-Pol auto-cleavage plotted against the CA/SP1 catalytic rate constant, with all other parameters fixed at their defaults. All points of an isocline yielded a fixed VMT (analogous to isoboles of combined drug doses of equal activity [30]). The isoclines are hyperbola-like functions with both vertical and horizontal asymptotes. This shape of the functions implies that for any given VMT, there is a minimum value for both parameters needed to achieve maturation within that given time; the vertical asymptotes indicate the minimum rates for CA/SP1 cleavage, the horizontal asymptotes indicate the minimal rate constants for Gag-Pol auto-cleavage. Close to the asymptotes, the corresponding slow rate becomes rate limiting, and very small changes in the limiting rate constant can only be compensated by large changes in the other parameter to maintain VMT. The default (empirical) parameter setting happens to fall in the regime where both parameters have comparable effect. This result indicates that small decreases in either rate constant (by drug or mutational effect) can be compensated by increases in the other parameter; however, compensation becomes increasingly difficult and eventually impossible as the affected rate parameter approaches its critical (asymptotic) value.

Even where compensation is possible in terms of VMT, the time course of Gag-Pol processing cannot be forced to return to the original behaviour. When a change in one of the dominant parameters is compensated by a change in the other to yield the same VMT, the time course of the process remains different from that obtained with the default parameters (Figure S3). “Complete” compensation of the time course would be possible only between parameters that have very similar local sensitivity functions [42]; however, the local sensitivity functions of the two dominant rate constants have different shapes (Figure S4). While some of the other parameters have similar sensitivity functions (for example, the sensitivity function of the Gag-Pol dissociation rate constant has similar shape to that of the catalytic rate constant of Gag-Pol auto-cleavage), the small magnitude of the effect of these on VMT precludes any meaningful compensation of changes in either of the dominant rate constants.

**Figure 6:**
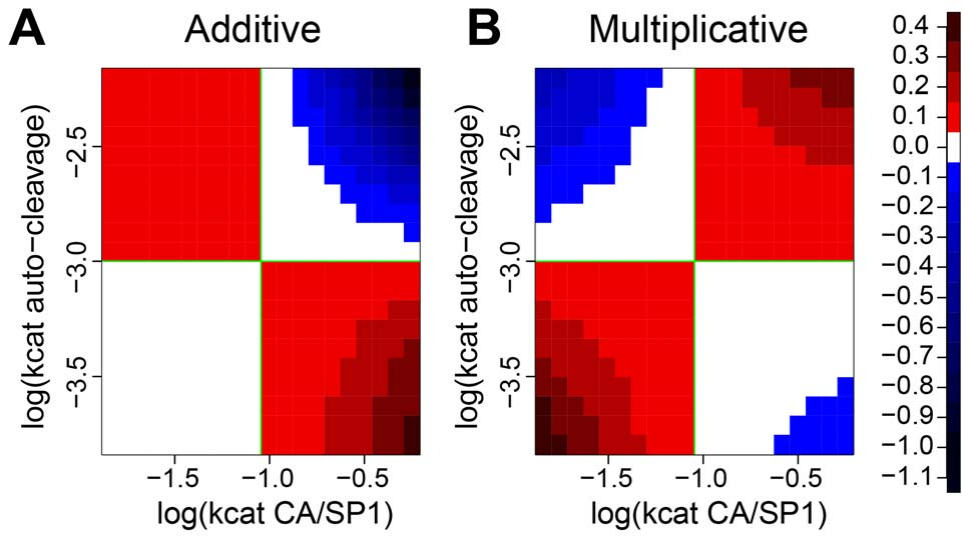
Accuracy of predicting combined parameter effects on VMT assuming additive (A) and multiplicative (B) interaction. Simulations were run with a geometric series of 20 values ranging from 0.16 to 6.25 times the default for both parameters, and with all 400 combinations of the variants. Under the additive model, predictions of combined effects were obtained by adding up absolute changes in VMT observed when changing each of the parameters separately; predictions under the multiplicative model were obtained by multiplying the relative (fold change) individual effects. Deviations (in minutes) from both predictions are plotted with color-coding; the dimensions of both parameters were s^−1^. The additive model performs better (smaller deviation) when both parameters are increased or both are decreased; the multiplicative model performs better when one parameter is increased and the other decreased.

We next investigated the potential interactions between the effects of the two dominant parameters when both are changed. In particular, we tested whether combined changes are characterized by either of two simple types of interaction: additive or multiplicative effects. We used the following simple definitions for the two types of interaction: using the notations VMT_def_, VMT_A_, VMT_B_ and VMT_AB_ to denote VMT obtained with the default parameters, with one, the other, or both of the parameters changed to a defined extent, we denote the absolute changes in VMT due to changes in each parameter with d_1_ = VMT_A_-VMT_def_ and d_2_ = VMT_B_-VMT_def_, and the fold changes with f_1_ = VMT_A_/VMT_def_ and f_2_ = VMT_B_/VMT_def_. The expected VMT when both parameters are changed is then VMT_AB_ = VMT_def_+d_1_+d_2_ under the additive model, and VMT_AB_ = VMT_def_*f_1_*f_2_ under the multiplicative model. We use the comparison with these two simple reference cases to illustrate the nature of the interaction depending on the direction of intervention and possible compensatory effects. We varied both parameters along a geometric series ranging from 0.16 to 6.25 times the default value, both separately and in all possible combinations. We used the results from the univariate series to predict the effect of combined changes assuming both additive and multiplicative effects, and tested the deviation of the simulations with combined changes from both predictions (Figure 6). We found that the additive model fits qualitatively better when both parameters are changed in the same direction (both increased or both decreased; Figure 6A), while the multiplicative model fits better when one parameter is increased and the other decreased (Figure 6B). Two scenarios might be most relevant biologically. First, compensatory mutations in one parameter might restore VMT in the presence of drugs or mutations that decrease the other parameter. In this case, one parameter is decreased and the other increased, which results in multiplicative interactions, consistent with the shape of the VMT isoclines (Figure 5). This implies that a given fold increase in one of the parameters can be compensated by a similar factor of decrease in the other parameter to restore the default VMT. Second, combinations of drugs might target both rates in concert, which corresponds to a decrease in both parameters. For this scenario, our results predict additive effects: the increase in VMT induced by such a combination can be approximated by the sum of the increases induced by monotherapy with the individual drugs. Synergistic drug effects are not expected.

### Protease inhibitor effect depends critically on inhibitor concentration and binding affinity

**Figure 7:**
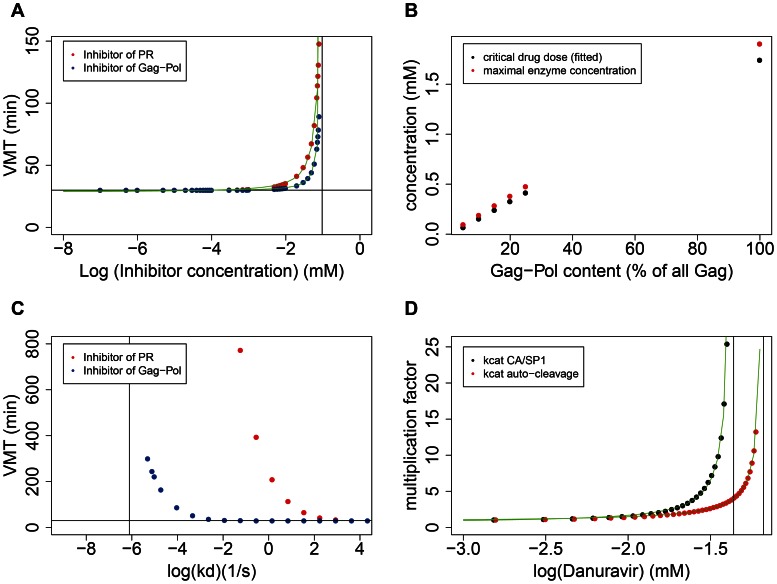
The effect of PR inhibitors on VMT. (A) The dependence of VMT on the concentration of PI that binds to mature PR (red symbols) and on the concentration of a hypothetical PI that binds to full-length Gag-Pol dimers and inhibits auto-cleavage (blue symbols). The binding rate constants of both PIs were parameterized with data estimated for the PR binding of darunavir. A hyperbola-like function of the form y = a+b/(c−x)^d^ could be fitted to the simulated VMT data, with a = 28.4 min, b = 8.75 min×mM, c = −0.91 mM, d = 1.53 for the inhibitor of PR, and with a = 29.4 min, b = 2.70 min×mM, c = −0.97 mM, d = 1.51 for the inhibitor of auto-cleavage. The vertical line indicates the theoretical maximum concentration of PR dimers at half of the initial concentration of Gag-Pol; the horizontal line indicates VMT in the absence of drug. (B) The effect of Gag-Pol content on critical drug concentration. Red dots indicate the theoretical maximum concentration of functional PR enzymes (half of Pol) at varied initial Gag-Pol content; black dots indicate the darunavir concentration required to delay virus maturation to VMT = 100 min in the model. (C) The dependence of VMT on the dissociation rate constant *k_d_* of an inhibitor of PR (red) and of an inhibitor of Gag-Pol (blue). The inhibitor of auto-cleavage requires greater binding affinity (lower log(k_d_)) to take effect. The vertical line indicates the estimated dissociation rate constant of darunavir. The horizontal line indicates VMT in the absence of drug; drug concentration was set to the possible maximal enzyme concentration (half of initial Gag-Pol) at 0.095 mM. (D) Isoclines of VMT = 30, in the plane of darunavir concentration against fold increase in the two catalytic parameters with the strongest effect on VMT. The same function as in (A) could be fitted with a = 0.57, b = 0.68 mM, c = −1.36 mM, d = 1.10 for the catalytic rate constant of CA/SP1 cleavage, and with a = 0.70, b = 0.66 mM, c = −1.18 mM, d = 0.94 for the catalytic rate constant of auto-cleavage. Vertical asymptotes (positioned by c values) show the limits on the compensation of drug effect by increased catalytic rates. In all panels, parameters were set as in Table 1 unless otherwise stated.

The modelling framework also allowed us to characterize the effect of PIs. As a test case, we selected darunavir, which is a potent inhibitor of HIV-1 PR [43], [44]. Figure 7A depicts the dependence of virion maturation time on the concentration of darunavir (red symbols) in the model. The response is very steep: VMT rises from the default value to infinity within about an order of magnitude range (∼0.01–0.1 mM) of the drug concentration, which is consistent with the steep dose response curves observed for PIs [27]. However, the PI concentration, where maturation is lost in the model is several orders of magnitude higher than the IC50 estimated for darunavir in infected cells *in vitro* [44], which calls for an explanation (see below). The vertical asymptote where maturation fails to occur is very close (at ∼0.12 mM) to the possible maximal concentration of PR dimers (at ∼0.095 mM; half of the initial Gag-Pol content), which implies that the majority of the enzyme needs to be blocked by the highly efficient inhibitor, if slower maturation still produces viable virions. This situation corresponds to the “critical subset” model of drug action [45], [46], which applies when enzyme function is insensitive to the drug concentration as long as a critical subset of enzyme molecules is unbound, but is lost quickly in the regime where the increasing drug concentration saturates the critical subset. Approximating the critical subset with the concentration of PR dimers that remain free in the presence of varied concentrations of the drug, the size of the subset is predicted to be around 30 PR dimers, if VMT = 60 min is required for viability, or around 15 dimers, if VMT>100 min is still tolerated (Figure S5). This result also predicts that the critical drug concentration needed to block virion maturation depends approximately linearly on the initial Gag-Pol content, and mutations affecting Gag-Pol frameshift will therefore have limited potential to compensate for the effect of PR inhibitors [47]. Figure 7B confirms this prediction.

While the shape of the dose response curve was consistent with the observations, there was a strong quantitative discrepancy between the model predictions and the empirical dose response observed *in vitro*. In the simulations, inhibition occurs where drug concentration is in the range of the maximal PR concentration (corresponding to half of the initial Pol content), which is in the ∼0.1 mM range. In contrast, *in vitro* experiments estimated an IC50 (half maximal inhibitory concentration) for darunavir in the nanomolar range [44], which implies a four to five orders of magnitude discrepancy between the estimates. The critical drug concentration in the model depends only on the assumption that a single drug molecule binds to and blocks a single PR dimer, and on the estimated Pol content (∼120 molecules) of a single virion. For a nanomolar drug concentration to take effect, a single molecule of drug should be able to block 10^4^–10^5^ PR dimers; in fact, a nanomolar concentration would imply that the average drug content of individual virions would be well below a single drug molecule per virion (which would correspond to a “concentration” of ∼1590 nM). That is, most virions would contain no drug molecules, unless there is drug enrichment. This result is independent of the details of the model, and the discrepancy highlights an important additional process, which has been overlooked previously. We propose that at low (nanomolar) drug concentrations in the medium, the critical drug concentration within the virion can be generated by diffusion and (near) irreversible binding to PR, which together result in the accumulation of drug from the surrounding medium to a form bound to PR in the virion. Darunavir has relatively high membrane permeability [48] and can even accumulate within cells [49], [50]. Assuming a drug concentration of 5 nM in the medium, and free diffusion of darunavir to the nascent virion, we calculate that the critical concentration (∼0.1 mM; a 2×10^4^ fold enrichment) required to block maturation can accumulate and bind PR in as little as a few minutes (Materials and methods). The rate limiting step is the association of the drug to PR, rather than diffusion to the virion, and the concentration of unbound PR is approximately halved per minute. Assuming that the critical subset comprises 1/2, 1/4, 1/8 of the total PR pool, it would thus take about 1, 2 or 3 minutes to accumulate the critical drug concentration needed to inhibit maturation. This simplistic calculation provides a lower boundary for the length of time when Gag-Pol processing is susceptible to the drug effect [51]. Note, however, that the beginning of the susceptible period might precede the budding of the virions, if the PR embedded in Gag-Pol can already be targeted by the PI within the cell. More realistic estimates for diffusion (that take into account possible barriers or the extensive binding of darunavir to proteins [48]) might in the future provide additional insight on the time window of susceptibility to PIs during the viral life cycle.

The model can also be used to predict the dependence of the drug effect on the binding affinity (parameterized by the dissociation rate constant) of the drug (Figure 7C). We found that the response to changes in the dissociation rate constant is similarly critical (steep) as to the concentration of the drug. Furthermore, the critical binding affinity required for the inhibition of maturation is several orders of magnitude lower than the estimated binding affinity of darunavir (and other potent drugs), which indicates that potent PIs operate with a broad “safety margin”. This is consistent with the observation that for darunavir a nearly 1000-fold decrease in binding affinity did not translate into a weaker antiviral activity [43], and might contribute to the relatively high genetic barrier of the drug. Implementing a hypothetical inhibitor that binds to full-length Gag-Pol to block the initial auto-cleavage produced dose response curves of very similar shapes; however, such inhibitors require much stronger binding affinity (close to that of darunavir) to take effect on VMT (Figure 7C: blue symbols), have weaker effect at the same fixed affinity and concentration (Figure 7A: blue symbols), and imply a smaller critical subset of unbound target molecules (Figure S5). This difference probably arises because unbound Gag-Pol molecules that undergo auto-cleavage generate active PR forms that can no longer be targeted by an (exclusive) inhibitor of Gag-Pol; in contrast, unbound PR remains a target for PIs until the cleavage of its internal cleavage site, upon which protease activity is lost.

We also tested the potential of the catalytic rate constants to compensate the effect of PIs (for example, by compensatory mutations in the cleavage sites [52], [53]). Figure 7D shows the compensation plots (isoclines of VMT = 30 min) of both dominant catalytic rates against the concentration of darunavir, demonstrating a limited potential for compensation. The vertical asymptote of the isocline for the CA/SP1 catalytic rate constant (at 10^−1.36^≈0.0436 mM) indicates that even an “infinite” catalytic rate could only compensate a drug dose of about 36% of the critical concentration (0.12 mM) that inhibits maturation completely, and *in vivo* drug levels with current dosing are likely to exceed the critical concentration considerably. The prediction of limited compensatory potential is in apparent contradiction with some empirical data that show clear compensation by substrate mutations in tissue culture and selection of such mutations *in vivo* [52], [53]); see the Discussion for a possible explanation.

### Initial inoculum of mature PR cannot substantially accelerate virion maturation

Finally, we investigated whether a small initial inoculum of mature PR would be able to accelerate virion maturation. Such an inoculum could potentially be derived either from the infecting virion or from Gag-Pol processing within the cell before virion assembly and budding. Figure S6 illustrates that a small initial inoculum has only a modest effect on the time to virion maturation; for example the addition of PR corresponding to 10% of Gag-Pol content reduces VMT from 30 min to about 25 min. A greater initial concentration of PR is unlikely at the beginning of the maturation process, given that premature proteolysis prior to confining the components in an assembling virion abolishes particle formation [54], which suggests that the bulk of proteolysis of virion associated proteins only occurs in the assembled virion (at or shortly after the time of budding). We therefore conclude that an initial inoculum of PR is unlikely to contribute substantially to proteolytic activity during maturation. The time scale of virion maturation therefore depends on Gag-Pol auto-cleavage within the virion, as has been assumed in our model. This result is also consistent with the observation that N-terminal cleavage follows first-order kinetics in protein concentration [8], [9], which implies that the dominant mechanism is intramolecular, rather than heteromolecular cleavage.

## Discussion

Our simulations of Gag-Pol processing are consistent with most of the known features of Gag-Pol processing (approximate time scale, order of release of final products), and can offer important insights into further details of the process that are not amenable to empirical study. In particular, we predicted the rate limiting steps in the maturation process, and our results suggest that the auto-cleavage of Gag-Pol dimers and the PR catalyzed cleavage at the CA/SP1 site are the most promising candidates for future drugs that would target individual steps of the proteolytic process. Importantly, bevirimat, the first clinically tested HIV-1 inhibitor that targets an individual cleavage site, as well as the chemically unrelated inhibitory compound PF-46396, affect cleavage at the CA/SP1 boundary [4], [5]. Unfortunately, drug combinations (or mutations) that inhibit or impair both dominant steps are not predicted to have a synergistic effect. Our model also provides a simple mechanistic explanation for the steep dose response curves associated with PIs [27], and highlights the importance of diffusion mediated drug accumulation in the virions (or in the infected cells before virion budding), which calls for further analyses. We demonstrated that the maturation process is robust with respect to variation in Gag content, and therefore also to stochastic biological variations in virion assembly, and showed that a small initial inoculum of mature PR is unable to “kick-start” the process. The model predicted that intermediate PR forms (with uncleaved C termini) may contribute substantially to proteolytic processing (this result clearly depends on the assumption that such intermediate forms have efficient catalytic activity [8], [55]). We also found that the self-cleavage of PR results in a loss of PR activity after the completion of maturation, which might be an evolutionary adaptation to avoid the loss of RT activity due to the cleavage of all p66 monomers and the possible loss of CA monomers due to cleavage of CA at non-canonical internal cleavage sites [17]. Finally, we were also able to predict the compensatory potential between drugs or mutations that affect the rate limiting steps or block PR activity. The compensatory potential of mutations affecting Gag-Pol frameshift [47] could also be investigated with the models.

While our “full model” of Gag-Pol processing provides valuable insights, this simplistic modelling approach clearly has a number of limitations. The use of mass action reaction kinetics assumes a well-mixed homogeneous system with concentrations described on a continuous scale, while both immature and mature virions have organized spatial structure [21], [40], [56], [57] and the number of enzyme and substrate molecules within a virion has a limited discrete scale of the order of hundreds and thousands, respectively [22], [31]. These constraints are likely to affect HIV-1 proteolytic processing and limit the validity of our model predictions [58]. To mitigate these limitations, the low number of interacting molecules could be addressed relatively simply by discrete stochastic modelling [59], while the introduction of explicit space would require a major re-structuring of the model, and might be a promising direction for further study.

Furthermore, the criteria that we used for maturation may have been incomplete. Our criteria for VMT involved the steps of proteolytic processing required for the morphological maturation of the capsid [25], [31], [35], but not the time needed for the assembly of the mature capsid. However, two observations indicate that assembly of the mature cone probably does not take very long: EM analyses have never revealed distinct maturation intermediates and in vitro assembly of CA seems to be very rapid following induction by high salt [60] (although assembly must be induced by a different trigger in vivo). Given these caveats, our definition of VMT based on processing criteria can be regarded as a lower bound but is likely to be a good approximation of the time to morphological maturation. However, there are probably other criteria for the viability (infectivity) of virions: each of the enzymes and structural proteins of the virus probably has a critical required count. Should quantitative data on other criteria be identified in the future, the model can easily be extended to accommodate the further requirements. We note also that the current limited maturation criteria are likely to introduce a bias on the set of parameters that can potentially influence VMT: the MA/CA, CA/SP1 and SP1/NC cleavage sites have direct effect on the molecular species that appear in the maturation criteria (of these, the processing of the MA/CA and SP1/NC cleavage sites occurs much faster than that of the CA/SP1 site, with k_cat_/K_M_ ratios of 45 and 74 vs. 9, which explains why only the latter has strong rate limiting effect), while Gag-Pol auto-cleavage is strictly needed to initiate processing, its rate therefore has a ubiquitous effect on the time course of the process. However, even with the current criteria, other rates (cleavage sites) could have strong indirect effects by competing for the available enzymes. The identity of the dominant rates is therefore not hard-wired in the structure of the model, but depends on the relative magnitude of all kinetic rates. For example, re-parameterizing the model with cleavage rates estimated with full-length Gag changes also the identity of rate limiting steps (see below).

To be able to use a consistent set of parameters, we had to rely on estimates obtained from the *in vitro* cleavage of oligopeptides containing individual cleavage sites in solution. However, flanking regions around the cleavage sites [20] and the extended context of variations in both the substrates and the enzyme [58] are likely to have an effect on the rates of cleavage by influencing the access of PR to the sites. In addition, the incorporation of Gag and Gag-Pol molecules in the strictly organized lattice architecture and the limited movement of molecules within the confined space of the virion are also likely to influence the effective cleavage rates. These effects may be particularly apparent regarding the removal of the spacer peptides by cleavage at the CA/SP1 and NC/SP2 processing site, respectively. Based on all available virological data, SP cleavage is considered to be a late event occurring after all other cleavages within Gag, which is not reflected in our simulations that were parameterized with peptide kinetic data (Figure 3B). There is indeed evidence that the CA/SP1 sites and NC/SP2 sites are sensitive to the lattice and RNA context. For example, the presence of uncleaved MA upstream [26], uncleaved NC downstream [11] or a proportion of uncleaved CA-SP1-NC molecules in the particle [35] reduces cleavage at the CA/SP1 site. Furthermore, exposure of the NC/SP2 site has been shown to be sensitive to the spatial organization of the RNA strands within the virion [61]. Such indirect effects might be responsible for the potency of the NC/SP2 cleavage site to affect the time scale of Gag processing and to compensate for inhibition by PIs [52], [53]), even though such an effect was not predicted in our simulations. Cleavage at this site was found to be much slower in the context of full-length Gag compared with that of short oligopeptides [11], which might increase the impact of this cleavage site on the time scale of virion maturation.

For Gag, quantitative empirical data are also available for the cleavage of the full polyprotein *in vitro* [11], and the relative rates estimated for some of the cleavage sites are different from those obtained in the context of oligopeptides, which might reflect the effect of flanking groups. We tested how an alternative set of cleavage rates in Gag, fitted to the empirical data on full-length Gag cleavage (adopted from [39]) affects the results of our model. The most important difference in the parameters is that CA/SP1 cleavage was much slower (*k_cat_* decreased, *K_M_* increased) compared with the estimates obtained with oligopeptides. As a result, virion maturation occurred much later, around ∼144 min (Figure S7), which is inconsistent with the fact that many released virions captured close to the cell by electron microscopy are mature and no morphological maturation intermediates are observed. Thus, although the true *in vivo* relative rates might be better approximated in the experiments that worked with full-length Gag instead of oligopeptides, the absolute rates of cleavage are likely to be much faster *in vivo* in the maturing virions. Furthermore, even full-length Gag *in vitro* is unlikely to reflect the spatial effects occurring in whole virions. However, the testing of this alternative parameter set yielded an important insight: the sensitivity of VMT to individual parameters also changes (Figure S8) compared with the default settings; a greater number of parameters have discernible effect on VMT with this parameter setting. This indicates that while our predictions on the identity of rate limiting steps are very robust as long as the dominant rate constants remain relatively close to their original values, large changes in these parameters (in this case in both CA/SP1 kinetic rate constants) may re-define the rate limiting steps of the process. In particular, it remains to be seen how well the currently used parameter estimates that were obtained with oligopeptide substrates reflect the real relative rates of cleavage in vivo: if future empirical work calls for major changes in the parameters, the simulation analyses should be repeated to accommodate the effect of the new estimates.

Functional p66/p51 heterodimers of reverse transcriptase (RT) decline to a relatively low level due to the cleavage of the p66 subunit into p51 and p15 fragments in the model. However, immunoblot analyses indicate that p66 and p51 persist in mature virions at approximately equal concentrations [62], [63], which suggests that some mechanism might protect RT from proteolysis *in vivo*. For example, the p51/p15 cleavage site might be cleaved preferentially in the context of p66 homodimers [38], [64], with greatly reduced cleavage both in monomers and in the functional heterodimer.

Finally, in all analyses we could only investigate VMT (the time to virion maturation) as a surrogate metric for the success of virion maturation. VMT predicted with the empirical set of kinetic parameters was consistent with current models of release and maturation [23], [36], [37], although some studies have estimated a longer time scale of several hours [65], while another retrovirus (Moloney Murine Leukemia Virus) seems to achieve partial maturation in as little as 5 minutes after release [66]. However, we have no direct data on how VMT translates to infectivity. As a rough approximation, maturation should be completed within the mean lifespan of virions, which is around ∼1 h in the blood plasma [67], and a few hours [68] or possibly even shorter [69] in the lymphoid tissues. Furthermore, cell-to-cell transmission (which plays a major role in HIV infection [70]) would also require rapid maturation after release; indeed, mature particles have been clearly observed in the virological synapse [71], although another study claimed that maturation only occurs upon endocytic uptake of the virus across the synapse [65]. However, further studies will be needed to better characterize VMT and its influence on replicative capacity of the viruses. In terms of the predicted drug effects, our results are likely to be robust due to the steep response curves: in the regime of the critical concentration and efficacy of drugs, VMT rises very sharply towards infinity (Figure 7), which firmly nails down the critical threshold required to block virus maturation effectively.

To our knowledge, our model is the first attempt to build and analyze a full model of proteolytic Gag-Pol processing in maturing HIV-1. Previous mathematical analyses have been restricted to approximating drug effect on series of subsequent cleavage reactions [72], without tracking individual substrates and products, while our previous analysis addressed the *in vitro* processing of Gag only [39]. The model developed here represents a major extension by taking into account all processing events for Gag and Gag-Pol, initial auto-cleavage and explicit enzyme dynamics. While subject to a number of limitations in its current form, this simulation framework provides a flexible platform to incorporate emerging empirical data in the future, including additional criteria for virus maturation or infectivity, measurements on the time course of proteolytic processing in whole virions (not available currently, but possibly amenable to emerging new technology), or more accurate data on the virion maturation time. Even current technology would allow the estimation of all kinetic rate constants (as in Table 1) for mutant PR forms, which could be used to generate detailed predictions on the influence of the mutations on the proteolytic process. The estimation of kinetic rates for combinations of PR forms, cleavage site variants and defined drug conditions could be used to characterize, and predict, complex evolutionary pathways under drug pressure. Quantitative data on the relationship between viral fitness and concentrations of functional RT or integrase could be used to predict the interactions of these drug classes with PR inhibitors [73] or PR mutations that impair the processing of these enzymes. Finally, the framework will be applicable to other viruses that rely on proteolytic processing of virion components, given that sufficient data on the respective cleavage rates are collected (for example, some kinetic data are available for the retroviruses feline immunodeficiency virus [74], Rous sarcoma virus [75] and murine leukaemia virus [76]). The publication of our full computer code under open access as a supplement to this paper will further facilitate the extension and broad application of this modelling framework in the future.

## Materials and Methods

### Notation of molecular species

Fully cleaved HIV-1 proteins and peptides are denoted by their standard abbreviations and nomenclature (see legend to Figure 1). Partially processed intermediates are denoted by concatenating the notations for the starting and the end fragment, for example, the intermediate species spanning CA-SP1-NC is denoted by CA.NC, the species spanning NC-TFP-p6^pol^-PR-p51 is denoted by NC.p51, etc. Dimers are denoted in the form M_1_dM_2_, where M_1,2_ denote the monomers; for example, PRdPR denotes the mature homodimer of fully liberated protease, MA.INdPR.IN denotes the initial product of the first step of Gag-Pol autocleavage.

### Model structure

**Figure 8:**
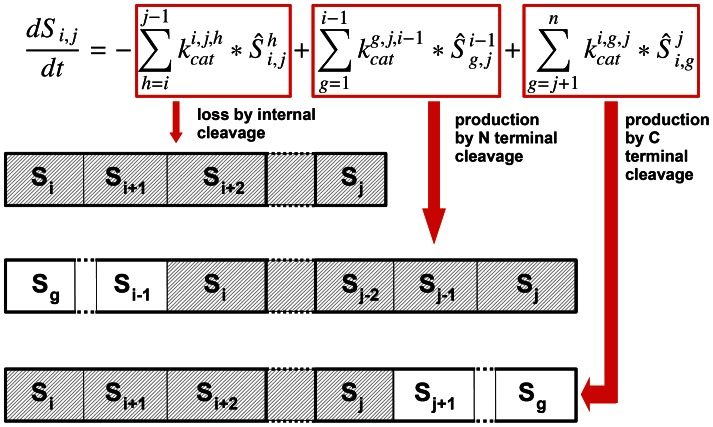
The rate of change in the concentration of a given fragment ***S_i,j_.*** Assuming quasi steady state for the enzyme-substrate complexes, 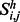, the rate of change consists of loss by cleavage at any of the internal cleavage sites, and production by “trimming” longer fragments that have an uncleaved site at either terminus.

Gag-Pol processing by the HIV-1 PR is a specific case of catalyzed competitive heteropolymer cleavage, for which we have developed a generic modelling framework in terms of Michaelis-Menten reaction kinetics under the quasi-steady-state (QSS) approximation [39]. In an abstract notation, let *S_i_* denote the *i^th^* monomer of the heteropolymer; *S_i,j_* a fragment spanning monomers *i* to *j*; and let the cleavage site *h* refer to the site C terminal to the *h^th^* monomer. Free protease can bind reversibly to any of the cleavage sites of a fragment such that *i≤h<j*, and let 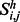 refer to an enzyme-substrate complex in which the enzyme is bound to cleavage site *h* within the fragment *S_i,j_*. Assuming Michaelis-Menten kinetics, the steady-state concentration of enzyme-substrate complexes can be written as 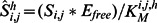, where *E_free_* denotes the concentration of free enzyme and 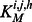 denotes the Michaelis-Menten constant of the cleavage of fragment *S_i,j_* at cleavage site *h*. Using *E_tot_ = E_free_+E_bound_* and summing over all distinct enzyme-substrate complexes we obtain: 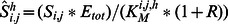, where 
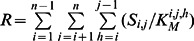
 with *n* denoting the length (number of distinct monomers) of the heteropolymer [39]. The rate of change in the concentration of *S_i,j_* can then be calculated as shown in Figure 8 (with 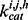 denoting the catalytic rate constant of the cleavage of fragment *S_i,j_* at cleavage site *h*). For example, the fragment CA.NC can be produced by the cleavage of MA.NC at the MA/CA cleavage site or by the cleavage of any CA.X fragment with X being a monomer downstream of NC at the NC/TFP cleavage site; and can be lost by internal cleavage at the CA/SP1 or the SP1/NC cleavage sites.

Proteolytic processing is initiated by the auto-cleavage of the full-length Gag-Pol dimer, which liberates the N-termini of both embedded PR domains in two steps [9], [28], [77]–[79]. PR activity is hampered primarily by the N-terminal flanking fragments of Gag-Pol [9], [55], [77], [80], and PR intermediates extended at the C terminus have been shown to have catalytic activity comparable to that of the mature enzyme [8], [55]. We therefore assumed that dimers of partially cleaved PR forms that have free N termini but uncleaved flanking groups at their C terminus already possess catalytic activity. For simplicity, we assumed that all active enzyme dimers have the same catalytic activity, and C terminal groups affect the efficiency of dimerization [81], rather than the rates of catalysis.

The model also tracked the formation and breakup of homo- and heterodimers of full-length Gag-Pol, intermediate and mature PR forms, and of p51/p66 heterodimers of the reverse transcriptase (RT). Because the quasi steady state of competing dimerization reactions cannot be computed in a closed form, we implemented a “hybrid” time scale in which enzyme-substrate complexes were kept in QSS within one time step, even though enzyme dimers were not. At the beginning of each time step, we summed up the level of enzymatic activity (total concentration of active enzyme dimers, *E_tot_*), and calculated the QSS for the enzyme-substrate complexes. The QSS concentrations of the complexes determined the rates of the net cleavage reactions within a time step. Enzyme dimers were allowed to form and dissociate within one time step, but the level of enzyme activity was only updated at the beginning of the next time step. Thus the iterative steps of our model were the following: 1) Sum up all active (intermediate and mature) enzyme dimers to calculate *E_tot_*; 2) calculate the QSS of enzyme-substrate complexes; 3) numerical integration of reactions involving the association and dissociation of enzyme dimers, the auto-cleavage reaction and the net cleavage reactions.

Because cleavage rates are typically estimated in the context of oligopeptides, the effect of flanking groups on cleavage could not be taken into account, that is, the same cleavage site was always cleaved with the same rate, irrespective of the substrate it was located in. We thus set 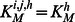 and 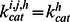 for all (*i,j*). All parameters are listed in Table 1.

For simplicity, dimers were assumed to be resistant to cleavage. In addition to the 11 canonical cleavage sites, we also implemented a non-canonical cleavage site within the mature PR monomer [15], which might be important for the auto-inactivation of proteolytic activity. Finally, we implemented two types of inhibitors: “classic” PIs that bind to active (intermediate and mature) PR dimers, and another hypothetical type that binds to dimers of full length Gag-Pol and blocks auto-cleavage (for simplicity, cross-inhibition of both types of dimers was not allowed).

### Implementation

The model was implemented in the C programming language, using an adaptive fifth order Cash-Krap Runge-Kutta algorithm for numerical integration of ordinary differential equations [82]. The full computer code of the simulations is available in the supplement Text S2. For the local sensitivity analyses, to be able to use the DASAC package [83], we have re-implemented the model in the Fortran programming language. We have verified that the two implementations yield consistent results.

### Global sensitivity analysis

The following 33 parameters were varied either individually or in combination: *k_cat_* and *K_M_* values for the 11 canonical cleavage sites and for the non-canonical internal cleavage site within PR; the rate of Gag-Pol auto-cleavage; the association and dissociation rates for the three classes of PR containing enzyme forms: mature PR, intermediate enzyme forms with cleaved N terminus, and full-length and intermediate forms with N terminal flanking groups; finally, the association and dissociation rates of RT heterodimers.

### Local sensitivity analysis

Local sensitivity analyses demonstrate how a small change in a particular parameter (from its default value) affects the time course of the concentrations during the simulations. The sensitivity of each variable (molecular species) with respect to each parameter is obtained as a function of time (along the time course of the simulated proteolytic process). We used half-normalized sensitivity functions that express the change in substrate concentrations as a function of relative change in a parameter (how much, in absolute units, does the concentration change, if we change the parameter by 1%). Local sensitivity analyses were performed with the help of the DASAC package [83] using the Fortran implementation of the model.

### Calculating the accumulation of darunavir by diffusion

Our simple calculation was based on diffusion from bulk medium to the surface of a sphere, assuming steady-state concentration gradient around the sphere and immediate uptake at the surface. Under these assumptions, total flux to the sphere (rate of accumulation) can be calculated as: 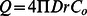, where *D* is the diffusion coefficient, *r* is the radius of the sphere and *C_0_* is the concentration in the bulk medium (at “infinite” distance from the sphere) [84]. The diffusion coefficient can be estimated by the Wilke-Chang method [85] as 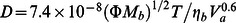, where Φ is a dimensionless association factor of the solvent (equal to 2.6 for water), *M_b_* is the molecular weight of the solvent (18.02 Da for water), *T* is the temperature in kelvins, *η_b_* is the viscosity of the solvent (0.862 cP for water at 300 K) and *V_a_* is the molar volume of the solute (408.4 cm^3^ for darunavir), which yields a diffusion coefficient of *D* = 4.78×10^−6^ cm^2^ s^−1^ for darunavir at T = 300 K in water. Given the estimated radius of a virion at *r* = 63 nm [22] and using a nanomolar concentration of *C_0_* = 5 nM for the bulk medium, the rate of accumulation of darunavir in the virion is estimated as *Q*≈2×10^−21^ mol/s. Considering an initial Gag-Pol content of 120 molecules per virion [22], the maximum amount of PR dimers is 60, or *E_max_* = 10^−22^ mol per virion. The time needed to accumulate an equal amount of drug molecules can then be calculated as *t_a_ = E_max_/Q* = 0.05 s. Assuming that this idealized diffusion process continuously replenishes the drug molecules that bind PR dimers within the virion, the drug concentration within the virion can be approximated with that in the bulk medium, and the binding kinetics of PR dimers by the drug can be characterized by the equation dE/dt = k_ass_*C_0_*E, where E denotes the concentration of unbound PR dimers and k_ass_ is the association rate of the drug. The time until a fraction *f* of all dimers remains unbound can then be calculated as −ln(f)/k_ass_*C_0_. Using the association rate of darunavir (Table 1) and *C_0_* = 5 nM, the concentration of unbound enzyme is approximately halved per minute (t_1/2_≈63s). Given that the dissociation rate of darunavir is <10^−6^/s (Table 1), the dissociation of drug-enzyme complexes can be neglected on the time scale of minutes.

### Statistics and function fitting

Statistical tests were performed using the R statistical environment [86]. Mathematical formulae were fitted to data points using the nls() function of R.

## Supporting Information

Figure S1 The time course of the major intermediates of simulated Gag (A) and Gag-Pol (B) processing.
(PDF)

Figure S2 The distribution of virion maturation times from 10,000 simulations with random parameter sets.
(PDF)

Figure S3 Compensation of virion maturation time (VMT) with different time courses of reaction products.
(PDF)

Figure S4 Half-normalized local sensitivity functions of the concentration of CA.SP1 with respect to selected parameters.
(PDF)

Figure S5 The size of the critical subset of PR or Gag-Pol dimers as a function of VMT required for viability. Simulations were run with different concentrations of two types of inhibitors binding either to mature PR (red symbols) or to full-length Gag-Pol dimers (blue symbols). The binding rate constants of both PIs were parameterized with data estimated for the PR binding of darunavir. The concentration of unbound enzyme was approximated by the total concentration of dimers minus the drug concentration; the axis to the right shows the corresponding numbers of dimers per virion. The amount of unbound enzyme characterizes the “critical subset” required to complete proteolytic processing within a given time (VMT). For example, the size of the subset is predicted to be around 30 PR dimers, if VMT = 60 min is required for viability, or around 15 dimers, if VMT>100 min is still tolerated. In the case of an inhibitor that binds Gag-Pol dimers, the critical subset of unbound target molecules was smaller than for the inhibitor of mature PR dimers at the same required VMT.
(PDF)

Figure S6 Adding an initial inoculum of mature protease results in modest decrease in VMT.
(PDF)

Figure S7 The time course of simulated Gag and Gag-Pol processing, using kinetic rate constants estimated based on full-length Gag cleavage. (A) Virus maturation time (VMT) (dashed red line in all panels) is still triggered by the decay of the CA.SP1 fragment (blue line; threshold of trans-dominant inhibition of particle maturation indicated by dashed horizontal line) and is not limited by the availability of liberated CA molecules (green line; threshold of one capsid unit corresponding to 1,500 CA molecules per particle is indicated by solid horizontal line), but occurs much later than with the default parameters. (B) Generation of catalytically active intermediate dimeric forms containing PR. (C) Decay of Gag substrate (black line) and accumulation of final Gag cleavage products. (D) Accumulation of final Pol cleavage products. (E) Enzyme concentrations and related metrics. The ratio PRdPR/E_tot_ indicates the relative contribution of mature PR dimers to the proteolytic activity. The ratio E_tot_/S_tot_ of the total concentration of active enzyme forms and the total concentration of uncleaved cleavage sites stays below one throughout the simulated time course, which justifies the use of Michaelis-Menten kinetics. E_tot_ – total proteolytic activity; S_tot_ – all uncleaved cleavage sites; IEF – all active intermediate enzyme (PR) forms; RT: p51/p66 heterodimer. All other dimers are indicated in the form M_1_dM_2_, where M_1,2_ are the monomers. Initial concentrations of Gag and Gag-Pol were set to reflect the quantities within a single virion; cleavage rates in Gag were parameterized as in [39]; all other parameters were set as in Table 1.
(PDF)

Figure S8 The effect of single parameter variation on VMT using an alternative set of kinetic rate constants.
(PDF)

Text S1 Estimation of catalytic rate constants.
(PDF)

Text S2 Computer code of simulations.
(ZIP)

## References

[1] Kohl NE, Emini EA, Schleif WA, Davis LJ, Heimbach JC, et al (1988) Active human immunodeficiency virus protease is required for viral infectivity. Proc Natl Acad Sci USA 85: 4686–4690. 3290901 10.1073/pnas.85.13.4686 PMC280500

[2] Anderson J, Schiffer C, Lee SK, Swanstrom R (2009) Viral protease inhibitors. Handb Exp Pharmacol 189: 85–110. 10.1007/978-3-540-79086-0_4 PMC7120715 19048198

[3] Wensing AM, van Maarseveen NM, Nijhuis M (2010) Fifteen years of HIV Protease Inhibitors: raising the barrier to resistance. Antiviral Res 85: 59–74. 19853627 10.1016/j.antiviral.2009.10.003

[4] Adamson CS, Salzwedel K, Freed EO (2009) Virus maturation as a new HIV-1 therapeutic target. Expert Opin Ther Targets 13: 895–908. 19534569 10.1517/14728220903039714 PMC2737327

[5] Waheed AA, Freed EO (2012) HIV type 1 Gag as a target for antiviral therapy. AIDS Res Hum Retroviruses 28: 54–75. 21848364 10.1089/aid.2011.0230 PMC3251841

[6] Debouck C, Gorniak JG, Strickler JE, Meek TD, Metcalf BW, et al (1987) Human immunodeficiency virus protease expressed in Escherichia coli exhibits autoprocessing and specific maturation of the gag precursor. Proc Natl Acad Sci USA 84: 8903–8906. 3321060 10.1073/pnas.84.24.8903 PMC299659

[7] Strickler JE, Gorniak J, Dayton B, Meek T, Moore M, et al (1989) Characterization and autoprocessing of precursor and mature forms of human immunodeficiency virus type 1 (HIV 1) protease purified from Escherichia coli. Proteins 6: 139–154. 2695927 10.1002/prot.340060205

[8] Louis JM, Nashed NT, Parris KD, Kimmel AR, Jerina DM (1994) Kinetics and mechanism of autoprocessing of human immunodeficiency virus type 1 protease from an analog of the Gag-Pol polyprotein. Proc Natl Acad Sci USA 91: 7970–7974. 8058744 10.1073/pnas.91.17.7970 PMC44526

[9] Louis JM, Wondrak EM, Kimmel AR, Wingfield PT, Nashed NT (1999) Proteolytic processing of HIV-1 protease precursor, kinetics and mechanism. J Biol Chem 274: 23437–23442. 10438521 10.1074/jbc.274.33.23437

[10] Beck ZQ, Morris GM, Elder JH (2002) Defining HIV-1 protease substrate selectivity. Curr Drug Targets Infect Disord 2: 37–50. 12462152 10.2174/1568005024605837

[11] Pettit SC, Henderson GJ, Schiffer CA, Swanstrom R (2002) Replacement of the P1 amino acid of human immunodeficiency virus type 1 Gag processing sites can inhibit or enhance the rate of cleavage by the viral protease. J Virol 76: 10226–10233. 12239298 10.1128/JVI.76.20.10226-10233.2002 PMC136535

[12] Barik D, Baumann WT, Paul MR, Novak B, Tyson JJ (2010) A model of yeast cell-cycle regulation based on multisite phosphorylation. Mol Syst Biol 6: 405. 20739927 10.1038/msb.2010.55 PMC2947364

[13] Tözsér J, Blaha I, Copeland TD, Wondrak EM, Oroszlan S (1991) Comparison of the HIV-1 and HIV-2 proteinases using oligopeptide substrates representing cleavage sites in Gag and Gag-Pol polyproteins. FEBS Lett 281: 77–80. 2015912 10.1016/0014-5793(91)80362-7

[14] Darke PL, Jordan SP, Hall DL, Zugay JA, Shafer JA, et al (1994) Dissociation and association of the HIV-1 protease dimer subunits: equilibria and rates. Biochemistry 33: 98–105. 8286367 10.1021/bi00167a013

[15] Mildner AM, Rothrock DJ, Leone JW, Bannow CA, Lull JM, et al (1994) The HIV-1 protease as enzyme and substrate: mutagenesis of autolysis sites and generation of a stable mutant with retained kinetic properties. Biochemistry 33: 9405–9413. 8068616 10.1021/bi00198a005

[16] Fehér A, Weber IT, Bagossi P, Boross P, Mahalingam B, et al (2002) Effect of sequence polymorphism and drug resistance on two HIV-1 Gag processing sites. Eur J Biochem 269: 4114–4120. 12180988 10.1046/j.1432-1033.2002.03105.x

[17] Tözsér J, Shulenin S, Kadas J, Boross P, Bagossi P, et al (2003) Human immunodeficiency virus type 1 capsid protein is a substrate of the retroviral proteinase while integrase is resistant toward proteolysis. Virology 310: 16–23. 12788626 10.1016/s0042-6822(03)00074-6

[18] Ludwig C, Leiherer A, Wagner R (2008) Importance of protease cleavage sites within and flanking human immunodeficiency virus type 1 transframe protein p6* for spatiotemporal regulation of protease activation. J Virol 82: 4573–4584. 18321978 10.1128/JVI.02353-07 PMC2293064

[19] Noel AF, Bilsel O, Kundu A, Wu Y, Zitzewitz JA, et al (2009) The folding free-energy surface of HIV-1 protease: insights into the thermodynamic basis for resistance to inhibitors. J Mol Biol 387: 1002–1016. 19150359 10.1016/j.jmb.2008.12.061 PMC2756696

[20] Breuer S, Sepulveda H, Chen Y, Trotter J, Torbett BE (2011) A cleavage enzyme-cytometric bead array provides biochemical profiling of resistance mutations in HIV-1 Gag and protease. Biochemistry 50: 4371–4381. 21452835 10.1021/bi200031m PMC3159576

[21] Briggs JA, Riches JD, Glass B, Bartonova V, Zanetti G, et al (2009) Structure and assembly of immature HIV. Proc Natl Acad Sci USA 106: 11090–11095. 19549863 10.1073/pnas.0903535106 PMC2700151

[22] Carlson LA, Briggs JA, Glass B, Riches JD, Simon MN, et al (2008) Three-dimensional analysis of budding sites and released virus suggests a revised model for HIV-1 morphogenesis. Cell Host Microbe 4: 592–599. 19064259 10.1016/j.chom.2008.10.013 PMC3454483

[23] Ivanchenko S, Godinez WJ, Lampe M, Krausslich HG, Eils R, et al (2009) Dynamics of HIV-1 assembly and release. PLoS Pathog 5: e1000652. 19893629 10.1371/journal.ppat.1000652 PMC2766258

[24] Jouvenet N, Simon SM, Bieniasz PD (2011) Visualizing HIV-1 assembly. J Mol Biol 410: 501–511. 21762796 10.1016/j.jmb.2011.04.062 PMC3144478

[25] Wright ER, Schooler JB, Ding HJ, Kieffer C, Fillmore C, et al (2007) Electron cryotomography of immature HIV-1 virions reveals the structure of the CA and SP1 Gag shells. EMBO J 26: 2218–2226. 17396149 10.1038/sj.emboj.7601664 PMC1852790

[26] de Marco A, Muller B, Glass B, Riches JD, Krausslich HG, et al (2010) Structural analysis of HIV-1 maturation using cryo-electron tomography. PLoS Pathog 6: e1001215. 21151640 10.1371/journal.ppat.1001215 PMC2999899

[27] Shen L, Peterson S, Sedaghat AR, McMahon MA, Callender M, et al (2008) Dose-response curve slope sets class-specific limits on inhibitory potential of anti-HIV drugs. Nat Med 14: 762–766. 18552857 10.1038/nm1777 PMC2743464

[28] Pettit SC, Clemente JC, Jeung JA, Dunn BM, Kaplan AH (2005) Ordered processing of the human immunodeficiency virus type 1 GagPol precursor is influenced by the context of the embedded viral protease. J Virol 79: 10601–10607. 16051852 10.1128/JVI.79.16.10601-10607.2005 PMC1182631

[29] Fitzgerald JB, Schoeberl B, Nielsen UB, Sorger PK (2006) Systems biology and combination therapy in the quest for clinical efficacy. Nat Chem Biol 2: 458–466. 16921358 10.1038/nchembio817

[30] Chou TC (2006) Theoretical basis, experimental design, and computerized simulation of synergism and antagonism in drug combination studies. Pharmacol Rev 58: 621–681. 16968952 10.1124/pr.58.3.10

[31] Briggs JA, Simon MN, Gross I, Krausslich HG, Fuller SD, et al (2004) The stoichiometry of Gag protein in HIV-1. Nat Struct Mol Biol 11: 672–675. 15208690 10.1038/nsmb785

[32] Accola MA, Hoglund S, Gottlinger HG (1998) A putative alpha-helical structure which overlaps the capsid-p2 boundary in the human immunodeficiency virus type 1 Gag precursor is crucial for viral particle assembly. J Virol 72: 2072–2078. 9499062 10.1128/jvi.72.3.2072-2078.1998 PMC109501

[33] Wiegers K, Rutter G, Kottler H, Tessmer U, Hohenberg H, et al (1998) Sequential steps in human immunodeficiency virus particle maturation revealed by alterations of individual Gag polyprotein cleavage sites. J Virol 72: 2846–2854. 9525604 10.1128/jvi.72.4.2846-2854.1998 PMC109729

[34] Checkley MA, Luttge BG, Soheilian F, Nagashima K, Freed EO (2010) The capsid-spacer peptide 1 Gag processing intermediate is a dominant-negative inhibitor of HIV-1 maturation. Virology 400: 137–144. 20172577 10.1016/j.virol.2010.01.028 PMC2838455

[35] Müller B, Anders M, Akiyama H, Welsch S, Glass B, et al (2009) HIV-1 Gag processing intermediates trans-dominantly interfere with HIV-1 infectivity. J Biol Chem 284: 29692–29703. 19666477 10.1074/jbc.M109.027144 PMC2785601

[36] Adamson CS, Freed EO (2007) Human immunodeficiency virus type 1 assembly, release, and maturation. Adv Pharmacol 55: 347–387. 17586320 10.1016/S1054-3589(07)55010-6

[37] Jouvenet N, Bieniasz PD, Simon SM (2008) Imaging the biogenesis of individual HIV-1 virions in live cells. Nature 454: 236–240. 18500329 10.1038/nature06998 PMC2708942

[38] Pettit SC, Lindquist JN, Kaplan AH, Swanstrom R (2005) Processing sites in the human immunodeficiency virus type 1 (HIV-1) Gag-Pro-Pol precursor are cleaved by the viral protease at different rates. Retrovirology 2: 66. 16262906 10.1186/1742-4690-2-66 PMC1291402

[39] Sadiq SK, Könnyü B, Müller V, Coveney PV (2011) Reaction kinetics of catalyzed competitive heteropolymer cleavage. J Phys Chem B 115: 11017–11027. 21823648 10.1021/jp206321b

[40] Briggs JA, Wilk T, Welker R, Krausslich HG, Fuller SD (2003) Structural organization of authentic, mature HIV-1 virions and cores. EMBO J 22: 1707–1715. 12660176 10.1093/emboj/cdg143 PMC152888

[41] Benjamin J, Ganser-Pornillos BK, Tivol WF, Sundquist WI, Jensen GJ (2005) Three-dimensional structure of HIV-1 virus-like particles by electron cryotomography. J Mol Biol 346: 577–588. 15670606 10.1016/j.jmb.2004.11.064 PMC6608732

[42] Lovrics A, Zsély IG, Csikász-Nagy A, Zádor J, Turányi T, et al (2008) Analysis of a budding yeast cell cycle model using the shapes of local sensitivity functions. International Journal of Chemical Kinetics 40: 710–720.

[43] Dierynck I, De Wit M, Gustin E, Keuleers I, Vandersmissen J, et al (2007) Binding kinetics of darunavir to human immunodeficiency virus type 1 protease explain the potent antiviral activity and high genetic barrier. J Virol 81: 13845–13851. 17928344 10.1128/JVI.01184-07 PMC2168871

[44] Koh Y, Matsumi S, Das D, Amano M, Davis DA, et al (2007) Potent inhibition of HIV-1 replication by novel non-peptidyl small molecule inhibitors of protease dimerization. J Biol Chem 282: 28709–28720. 17635930 10.1074/jbc.M703938200

[45] Shen L, Rabi SA, Sedaghat AR, Shan L, Lai J, et al (2011) A critical subset model provides a conceptual basis for the high antiviral activity of major HIV drugs. Sci Transl Med 3: 91ra63. 10.1126/scitranslmed.3002304 PMC3488347 21753122

[46] Perelson AS, Deeks SG (2011) Drug effectiveness explained: the mathematics of antiviral agents for HIV. Sci Transl Med 3: 91ps30. 10.1126/scitranslmed.3002656 PMC3236558 21753120

[47] Doyon L, Payant C, Brakier-Gingras L, Lamarre D (1998) Novel Gag-Pol frameshift site in human immunodeficiency virus type 1 variants resistant to protease inhibitors. J Virol 72: 6146–6150. 9621079 10.1128/jvi.72.7.6146-6150.1998 PMC110421

[48] Back D, Sekar V, Hoetelmans RM (2008) Darunavir: pharmacokinetics and drug interactions. Antivir Ther 13: 1–13. 18389894

[49] Kwan WS, Janneh O, Hartkoorn R, Chandler B, Khoo S, et al (2009) Intracellular ‘boosting’ of darunavir using known transport inhibitors in primary PBMC. Br J Clin Pharmacol 68: 375–380. 19740394 10.1111/j.1365-2125.2009.03462.x PMC2766476

[50] Jackson A, Watson V, Back D, Khoo S, Liptrott N, et al (2011) Plasma and intracellular pharmacokinetics of darunavir/ritonavir once daily and raltegravir once and twice daily in HIV-infected individuals. J Acquir Immune Defic Syndr 58: 450–457. 21926632 10.1097/QAI.0b013e3182364c67 PMC3594701

[51] Sedaghat AR, Wilke CO (2011) Kinetics of the viral cycle influence pharmacodynamics of antiretroviral therapy. Biol Direct 6: 42. 21910865 10.1186/1745-6150-6-42 PMC3203257

[52] Nijhuis M, van Maarseveen NM, Lastere S, Schipper P, Coakley E, et al (2007) A novel substrate-based HIV-1 protease inhibitor drug resistance mechanism. PLoS Med 4: e36. 17227139 10.1371/journal.pmed.0040036 PMC1769415

[53] van Maarseveen NM, Andersson D, Lepsik M, Fun A, Schipper PJ, et al (2012) Modulation of HIV-1 Gag NC/p1 cleavage efficiency affects protease inhibitor resistance and viral replicative capacity. Retrovirology 9: 29. 22462820 10.1186/1742-4690-9-29 PMC3349524

[54] Kräusslich HG (1991) Human immunodeficiency virus proteinase dimer as component of the viral polyprotein prevents particle assembly and viral infectivity. Proc Natl Acad Sci USA 88: 3213–3217. 2014242 10.1073/pnas.88.8.3213 PMC51416

[55] Wondrak EM, Nashed NT, Haber MT, Jerina DM, Louis JM (1996) A transient precursor of the HIV-1 protease. Isolation, characterization, and kinetics of maturation. J Biol Chem 271: 4477–4481. 8626801 10.1074/jbc.271.8.4477

[56] Bharat TA, Davey NE, Ulbrich P, Riches JD, de Marco A, et al (2012) Structure of the immature retroviral capsid at 8 A resolution by cryo-electron microscopy. Nature 487: 385–389. 22722831 10.1038/nature11169

[57] Briggs JA, Krausslich HG (2011) The molecular architecture of HIV. J Mol Biol 410: 491–500. 21762795 10.1016/j.jmb.2011.04.021

[58] Lee SK, Potempa M, Swanstrom R (2012) The choreography of HIV-1 proteolytic processing and virion assembly. J Biol Chem 287: 40867–40874. 23043111 10.1074/jbc.R112.399444 PMC3510790

[59] Gillespie DT (2007) Stochastic simulation of chemical kinetics. Annu Rev Phys Chem 58: 35–55. 17037977 10.1146/annurev.physchem.58.032806.104637

[60] Douglas CC, Thomas D, Lanman J, Prevelige PE Jr (2004) Investigation of N-terminal domain charged residues on the assembly and stability of HIV-1 CA. Biochemistry 43: 10435–10441. 15301542 10.1021/bi049359g

[61] Mirambeau G, Lyonnais S, Coulaud D, Hameau L, Lafosse S, et al (2007) HIV-1 protease and reverse transcriptase control the architecture of their nucleocapsid partner. PLoS One 2: e669. 17712401 10.1371/journal.pone.0000669 PMC1940317

[62] di Marzo Veronese F, Copeland TD, DeVico AL, Rahman R, Oroszlan S, et al (1986) Characterization of highly immunogenic p66/p51 as the reverse transcriptase of HTLV-III/LAV. Science 231: 1289–1291. 2418504 10.1126/science.2418504

[63] Welker R, Kottler H, Kalbitzer HR, Krausslich HG (1996) Human immunodeficiency virus type 1 Nef protein is incorporated into virus particles and specifically cleaved by the viral proteinase. Virology 219: 228–236. 8623533 10.1006/viro.1996.0240

[64] Sluis-Cremer N, Arion D, Abram ME, Parniak MA (2004) Proteolytic processing of an HIV-1 pol polyprotein precursor: insights into the mechanism of reverse transcriptase p66/p51 heterodimer formation. Int J Biochem Cell Biol 36: 1836–1847. 15183348 10.1016/j.biocel.2004.02.020

[65] Dale BM, McNerney GP, Thompson DL, Hubner W, de Los Reyes K, et al (2011) Cell-to-cell transfer of HIV-1 via virological synapses leads to endosomal virion maturation that activates viral membrane fusion. Cell Host Microbe 10: 551–562. 22177560 10.1016/j.chom.2011.10.015 PMC3278276

[66] Fu W, Rein A (1993) Maturation of dimeric viral RNA of Moloney murine leukemia virus. J Virol 67: 5443–5449. 8350405 10.1128/jvi.67.9.5443-5449.1993 PMC237946

[67] Ramratnam B, Bonhoeffer S, Binley J, Hurley A, Zhang L, et al (1999) Rapid production and clearance of HIV-1 and hepatitis C virus assessed by large volume plasma apheresis. Lancet 354: 1782–1785. 10577640 10.1016/S0140-6736(99)02035-8

[68] Müller V, Maree AF, De Boer RJ (2001) Release of virus from lymphoid tissue affects human immunodeficiency virus type 1 and hepatitis C virus kinetics in the blood. J Virol 75: 2597–2603. 11222682 10.1128/JVI.75.6.2597-2603.2001 PMC115882

[69] De Boer RJ, Ribeiro RM, Perelson AS (2010) Current estimates for HIV-1 production imply rapid viral clearance in lymphoid tissues. PLoS Comput Biol 6: e1000906. 20824126 10.1371/journal.pcbi.1000906 PMC2932679

[70] Sattentau QJ (2010) Cell-to-Cell Spread of Retroviruses. Viruses 2: 1306–1321. 21994681 10.3390/v2061306 PMC3185708

[71] Jolly C, Kashefi K, Hollinshead M, Sattentau QJ (2004) HIV-1 cell to cell transfer across an Env-induced, actin-dependent synapse. J Exp Med 199: 283–293. 14734528 10.1084/jem.20030648 PMC2211771

[72] Rasnick D (1997) Kinetics analysis of consecutive HIV proteolytic cleavages of the Gag-Pol polyprotein. J Biol Chem 272: 6348–6353. 9045655

[73] Snyder S, D'Argenio DZ, Weislow O, Bilello JA, Drusano GL (2000) The triple combination indinavir-zidovudine-lamivudine is highly synergistic. Antimicrob Agents Chemother 44: 1051–1058. 10722511 10.1128/aac.44.4.1051-1058.2000 PMC89812

[74] Lin YC, Brik A, de Parseval A, Tam K, Torbett BE, et al (2006) Altered gag polyprotein cleavage specificity of feline immunodeficiency virus/human immunodeficiency virus mutant proteases as demonstrated in a cell-based expression system. J Virol 80: 7832–7843. 16873240 10.1128/JVI.00374-06 PMC1563824

[75] Vana ML, Chen A, Boross P, Weber I, Colman D, et al (2005) Mutations affecting cleavage at the p10-capsid protease cleavage site block Rous sarcoma virus replication. Retrovirology 2: 58. 16188035 10.1186/1742-4690-2-58 PMC1262776

[76] Menendez-Arias L, Weber IT, Soss J, Harrison RW, Gotte D, et al (1994) Kinetic and modeling studies of subsites S4-S3' of Moloney murine leukemia virus protease. J Biol Chem 269: 16795–16801. 8207003

[77] Louis JM, Clore GM, Gronenborn AM (1999) Autoprocessing of HIV-1 protease is tightly coupled to protein folding. Nat Struct Biol 6: 868–875. 10467100 10.1038/12327

[78] Pettit SC, Everitt LE, Choudhury S, Dunn BM, Kaplan AH (2004) Initial cleavage of the human immunodeficiency virus type 1 GagPol precursor by its activated protease occurs by an intramolecular mechanism. J Virol 78: 8477–8485. 15280456 10.1128/JVI.78.16.8477-8485.2004 PMC479095

[79] Sadiq SK, Noe F, De Fabritiis G (2012) Kinetic characterization of the critical step in HIV-1 protease maturation. Proc Natl Acad Sci USA 109: 20449–20454. 23184967 10.1073/pnas.1210983109 PMC3528573

[80] Chatterjee A, Mridula P, Mishra RK, Mittal R, Hosur RV (2005) Folding regulates autoprocessing of HIV-1 protease precursor. J Biol Chem 280: 11369–11378. 15632156 10.1074/jbc.M412603200

[81] Wondrak EM, Louis JM (1996) Influence of flanking sequences on the dimer stability of human immunodeficiency virus type 1 protease. Biochemistry 35: 12957–12962. 8841142 10.1021/bi960984y

[82] Press WH, Teukolsky SA, Vetterling WT, Flannery BT (1992) Numerical Recipes in C: the art of scientific computing. New York: Cambridge University Press.

[83] Caracotsios M, Stewart WE (1985) Sensitivity analysis of initial value problems with mixed odes and algebraic equations. Computers & Chemical Engineering 9: 359–365.

[84] Crank J (1975) The Mathematics of Diffusion. Oxford: Clarendon Press.

[85] Wilke CR, Chang P (1955) Correlation of diffusion coefficients in dilute solutions. AICHE J 1: 264–270.

[86] R Core Team (2012) R: A Language and Environment for Statistical Computing. Vienna: R Foundation for Statistical Computing.

[87] Divita G, Restle T, Goody RS (1993) Characterization of the dimerization process of HIV-1 reverse transcriptase heterodimer using intrinsic protein fluorescence. FEBS Lett 324: 153–158. 7685295 10.1016/0014-5793(93)81383-b

