# Supplementary figures and images for: Gag-Pol Processing during HIV-1 Virion Maturation: A Systems Biology Approach
Source: PLoS Comput Biol. 2013 Jun 6;9(6):e1003103. doi: 10.1371/journal.pcbi.1003103 (PMC3675044; doi:10.1371/journal.pcbi.1003103)

**A**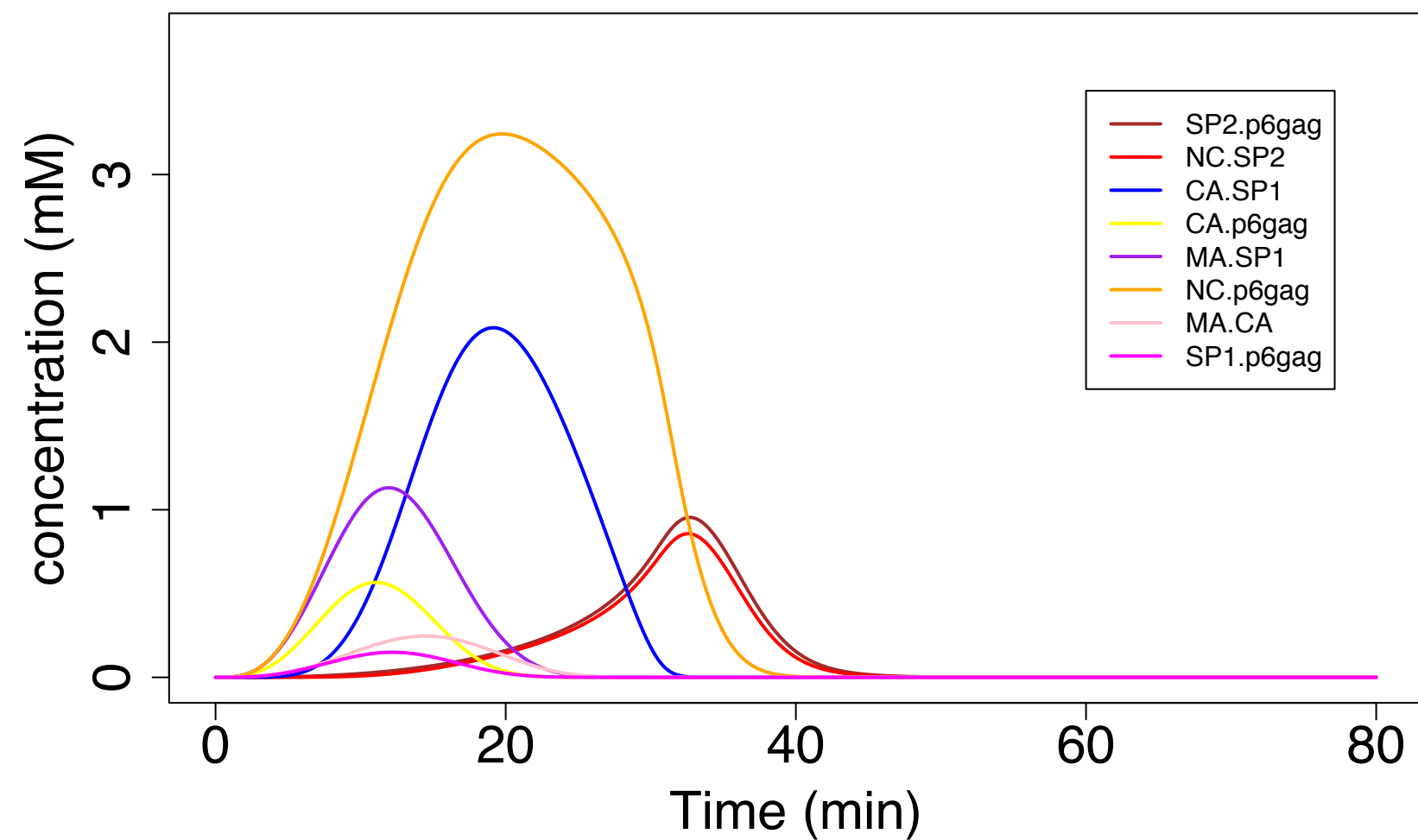**B**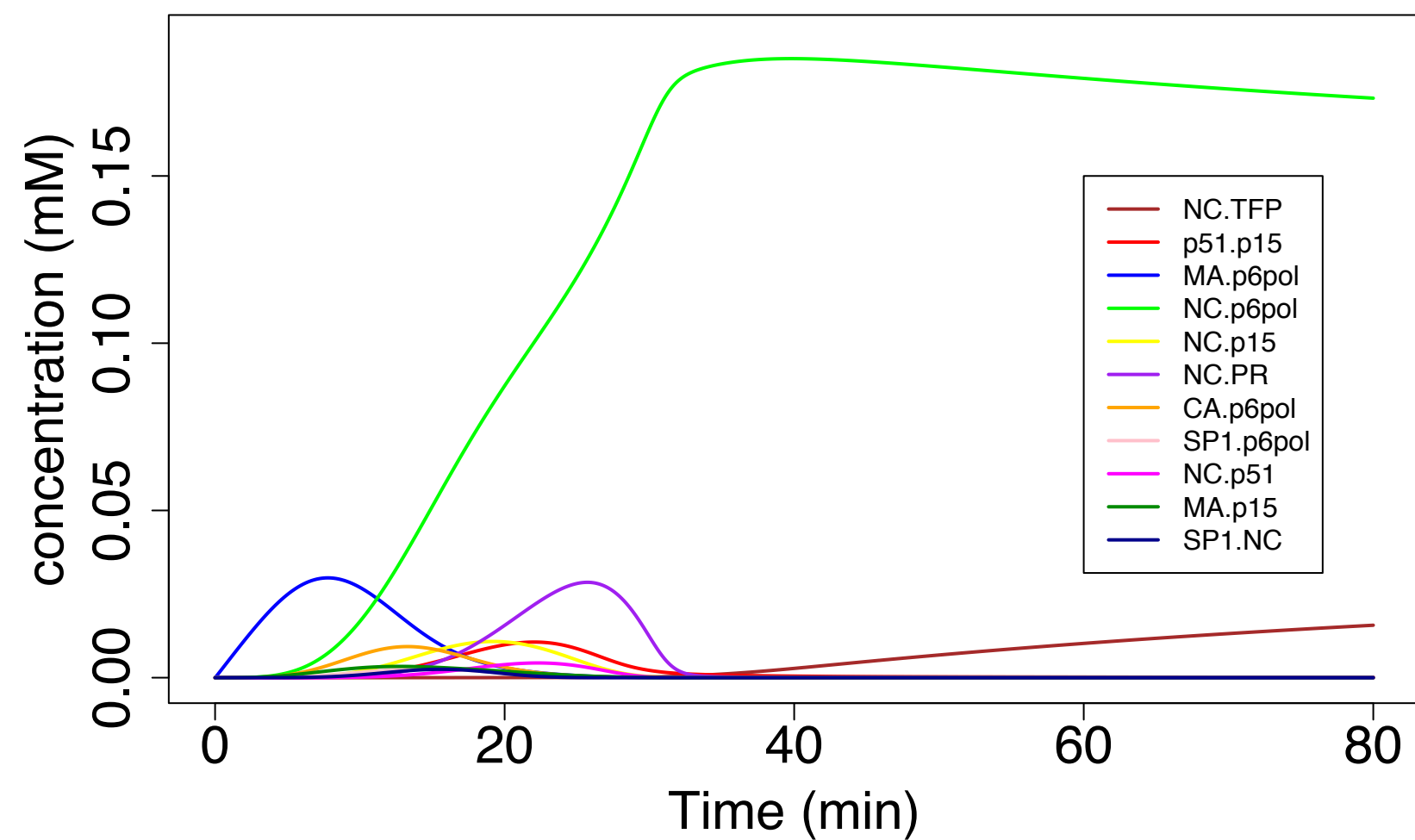

Supplement: Figure S1 — The time course of the major intermediates of simulated Gag (A) and Gag-Pol (B) processing. (PDF) [file pcbi.1003103.s001.pdf]

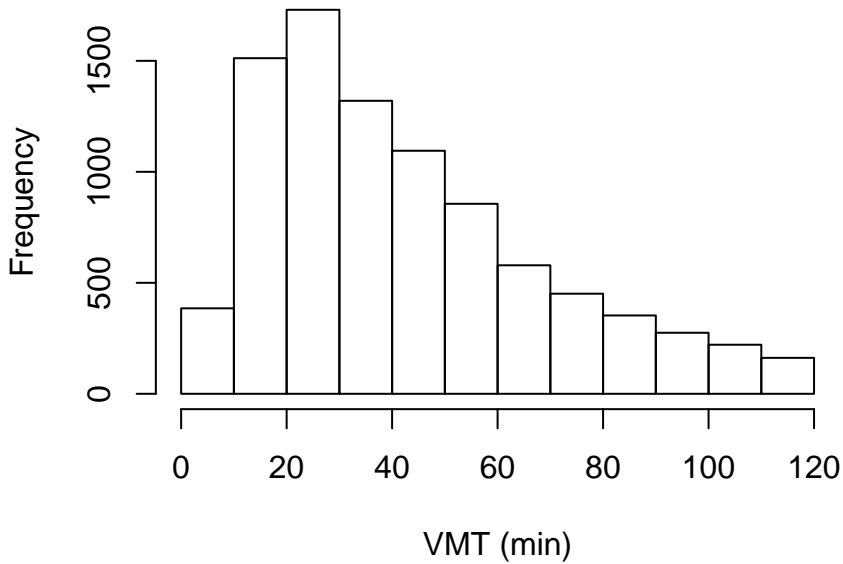

Supplement: Figure S2 — The distribution of virion maturation times from 10,000 simulations with random parameter sets. (PDF) [file pcbi.1003103.s002.pdf]

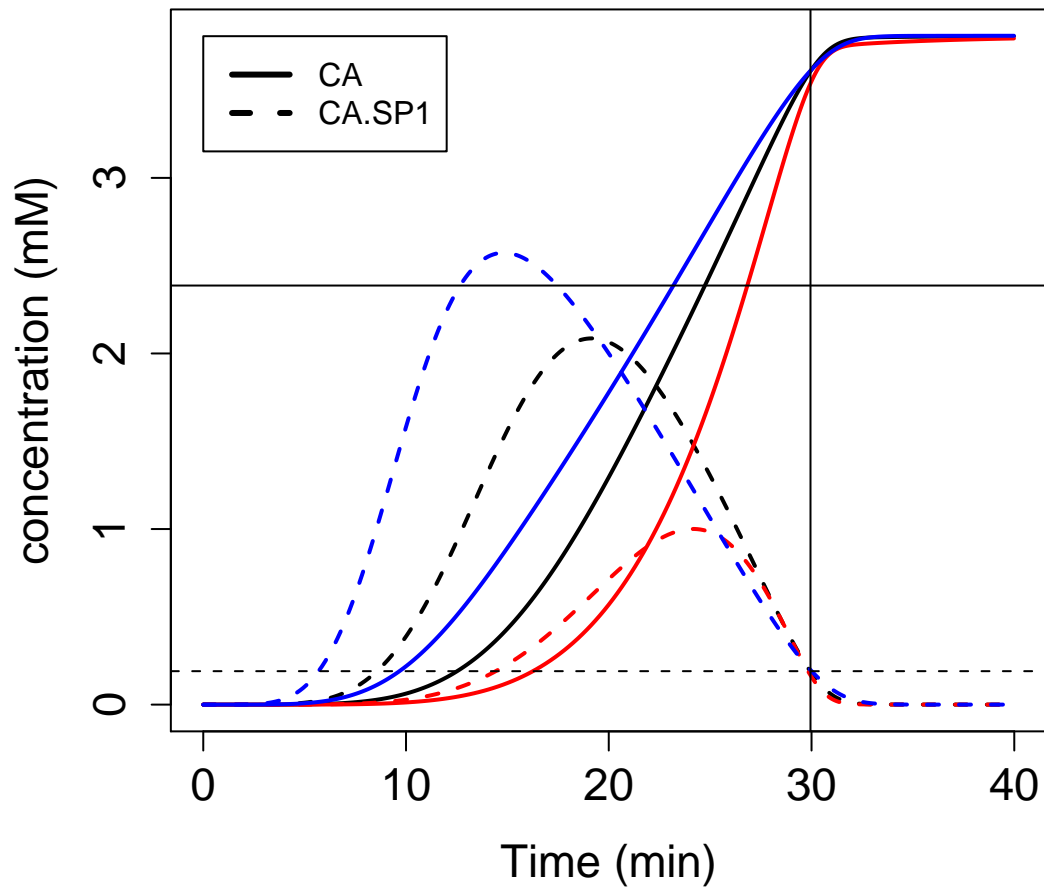

Supplement: Figure S3 — Compensation of virion maturation time (VMT) with different time courses of reaction products. (PDF) [file pcbi.1003103.s003.pdf]

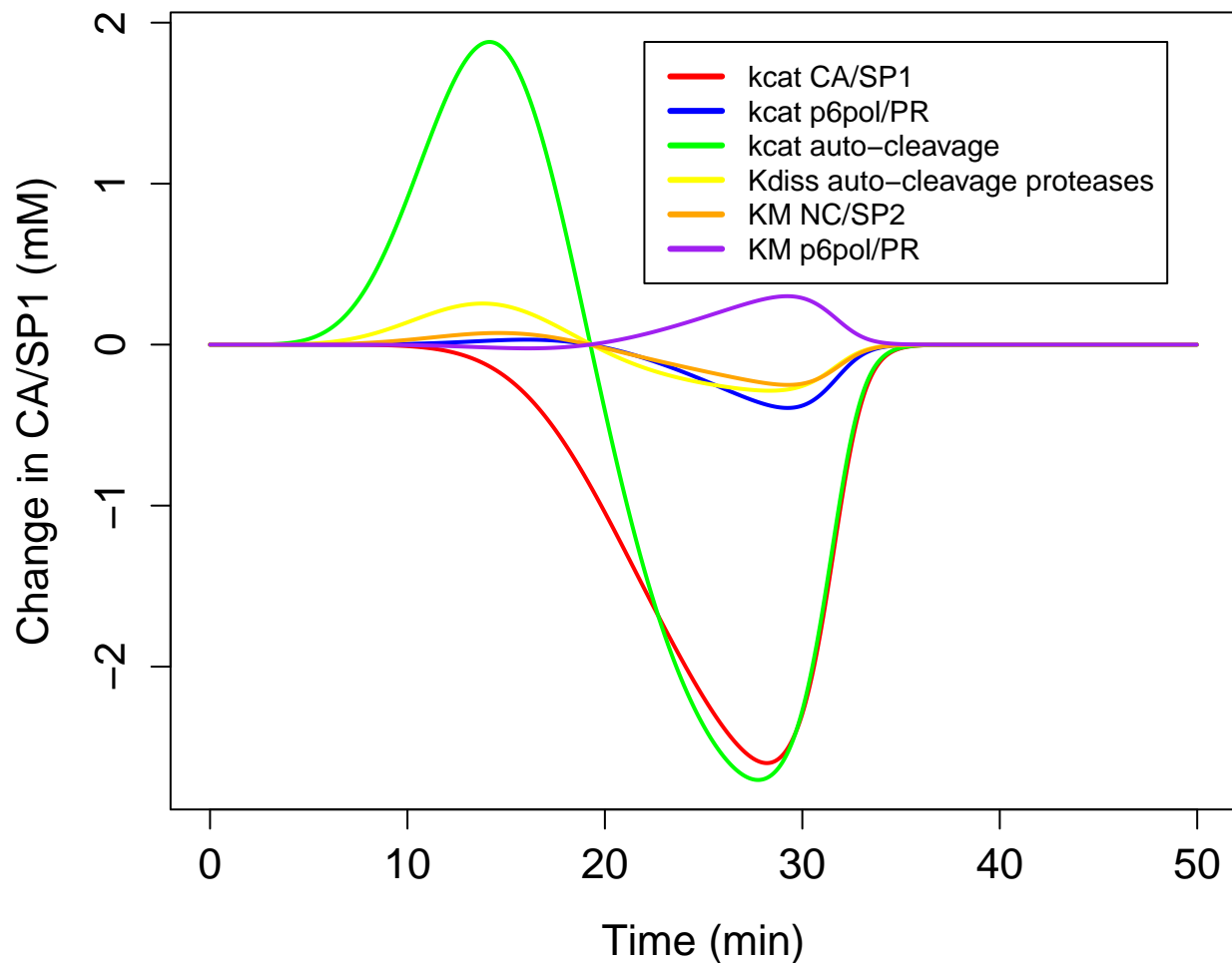

Supplement: Figure S4 — Half-normalized local sensitivity functions of the concentration of CA.SP1 with respect to selected parameters. (PDF) [file pcbi.1003103.s004.pdf]

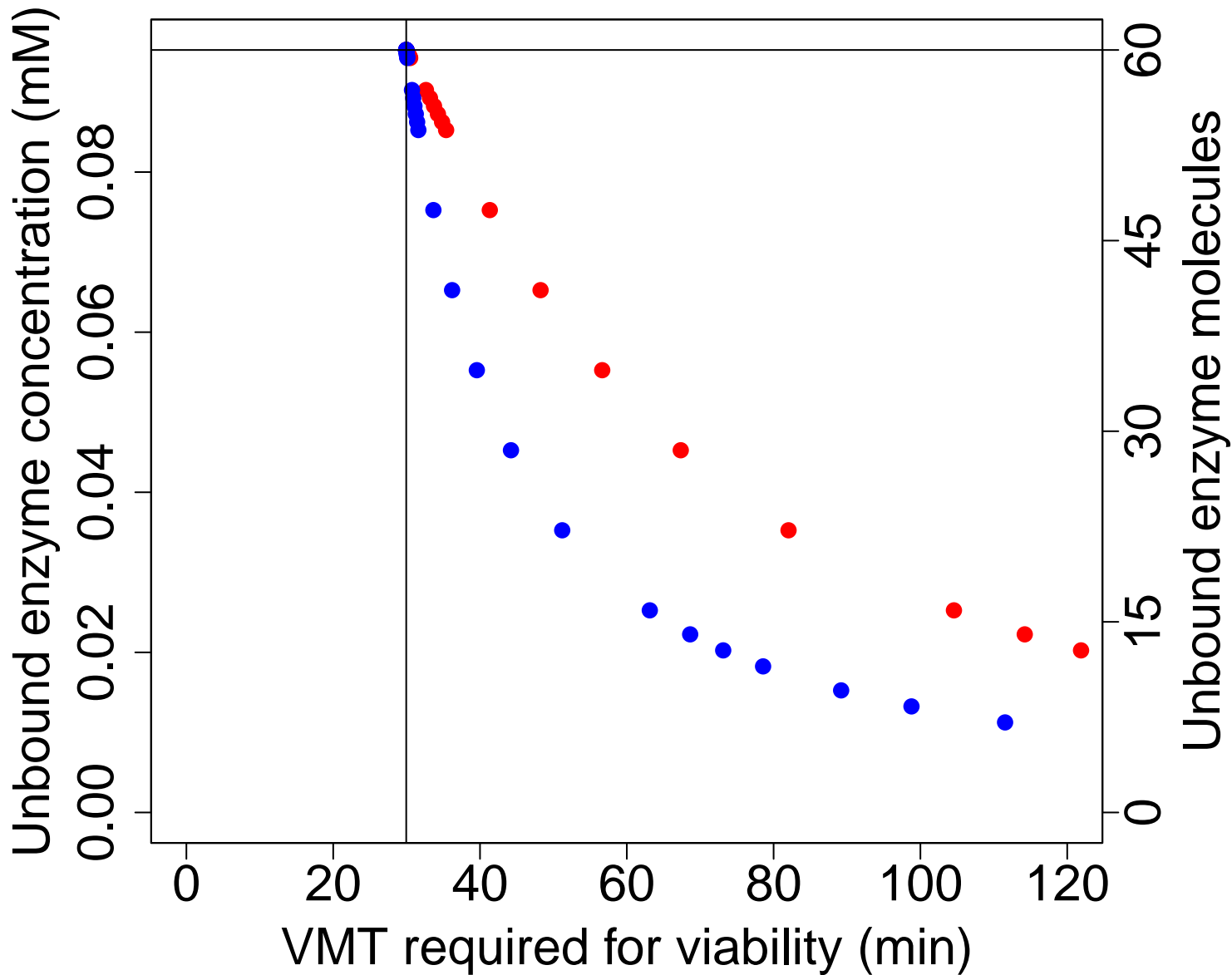

Supplement: Figure S5 — The size of the critical subset of PR or Gag-Pol dimers as a function of VMT required for viability. Simulations were run with different concentrations of two types of inhibitors binding either to mature PR (red symbols) or to full-length Gag-Pol dimers (blue symbols). The binding rate constants of both PIs were parameterized with data estimated for the PR binding of darunavir. The concentration of unbound enzyme was approximated by the total concentration of dimers minus the drug concentration; the axis to the right shows the corresponding numbers of dimers per virion. The amount of unbound enzyme characterizes the “critical subset” required to complete proteolytic processing within a given time (VMT). For example, the size of the subset is predicted to be around 30 PR dimers, if VMT = 60 min is required for viability, or around 15 dimers, if VMT>100 min is still tolerated. In the case of an inhibitor that binds Gag-Pol dimers, the critical subset of unbound target molecules was smaller than for the inhibitor of mature PR dimers at the same required VMT. (PDF) [file pcbi.1003103.s005.pdf]

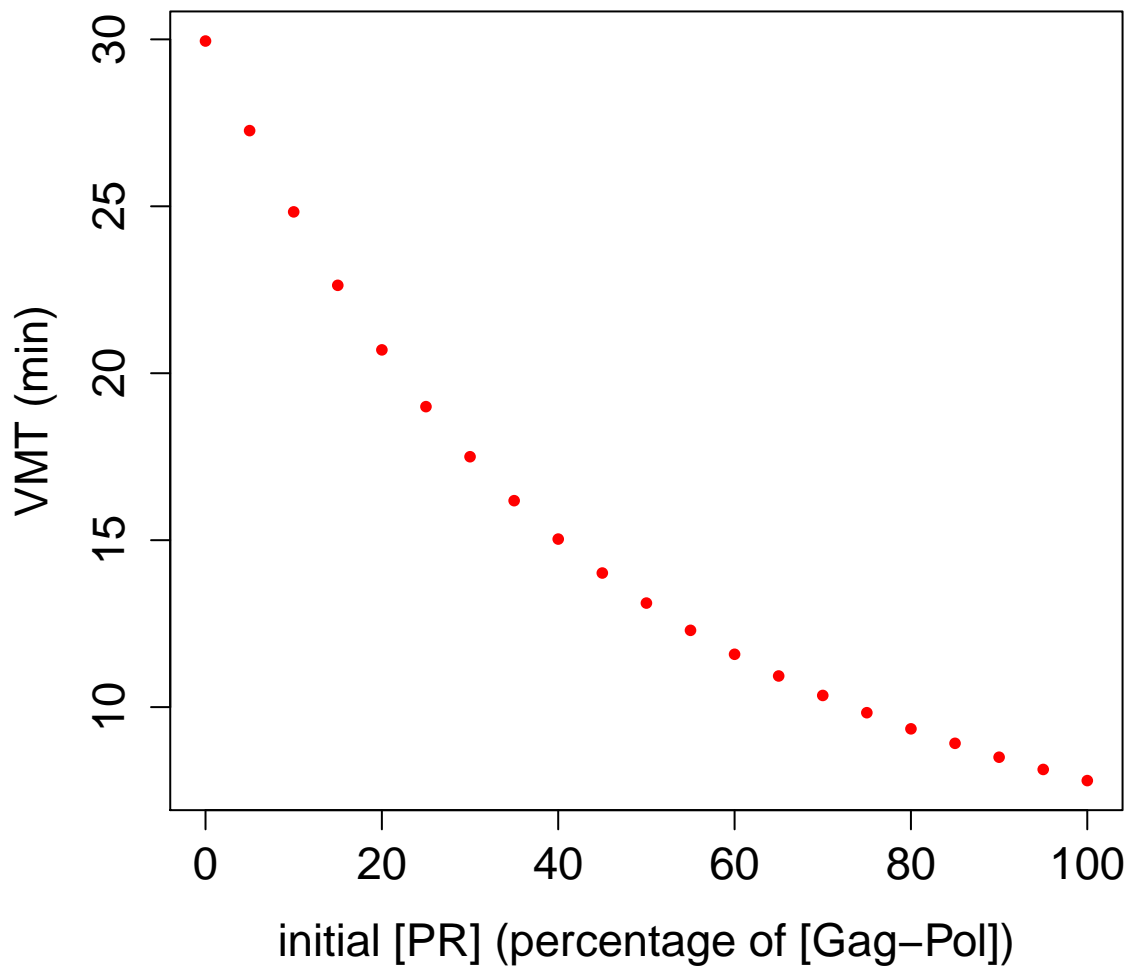

Supplement: Figure S6 — Adding an initial inoculum of mature protease results in modest decrease in VMT. (PDF) [file pcbi.1003103.s006.pdf]

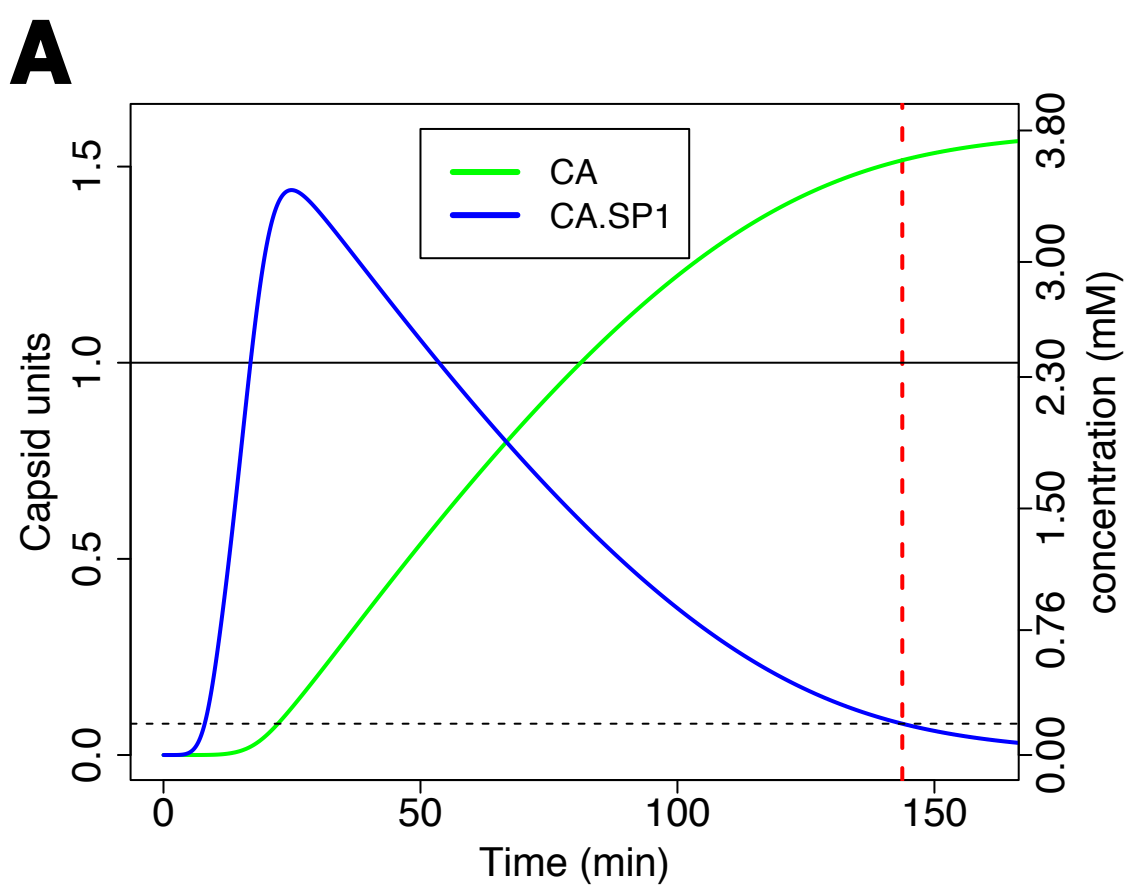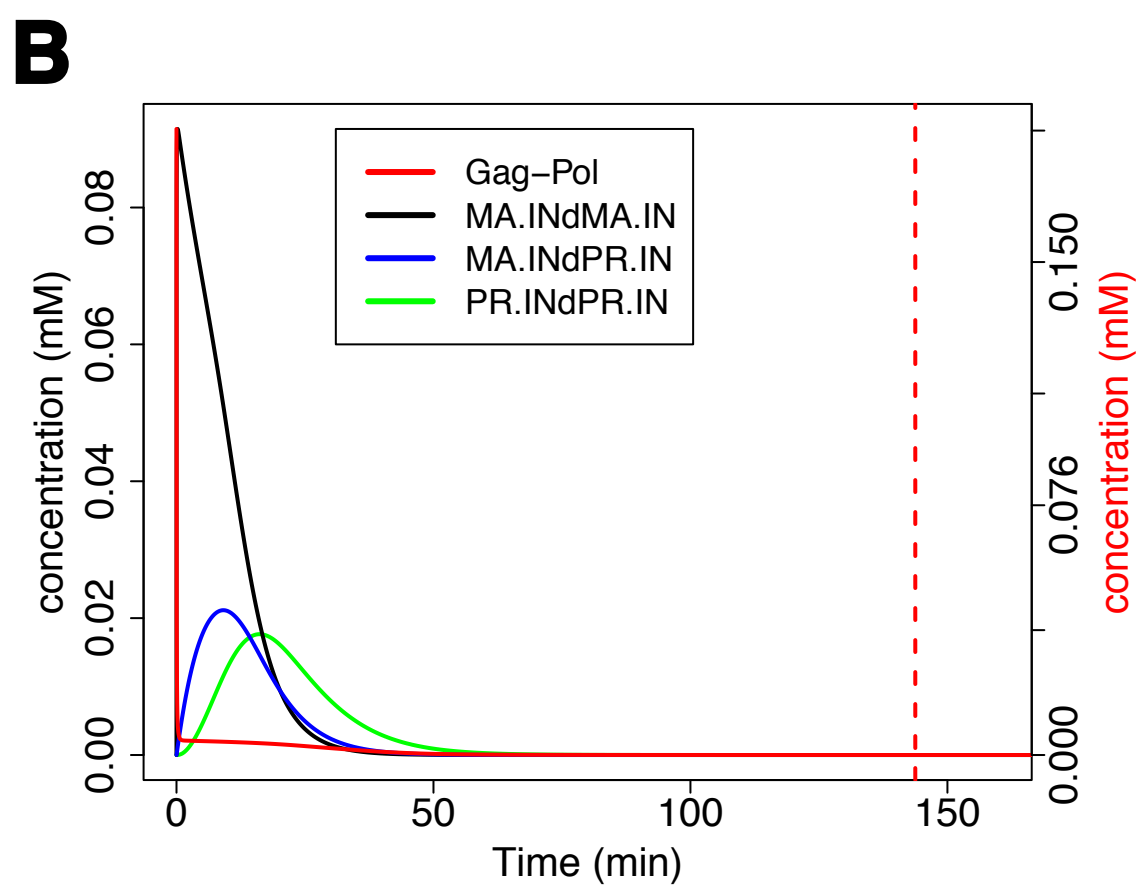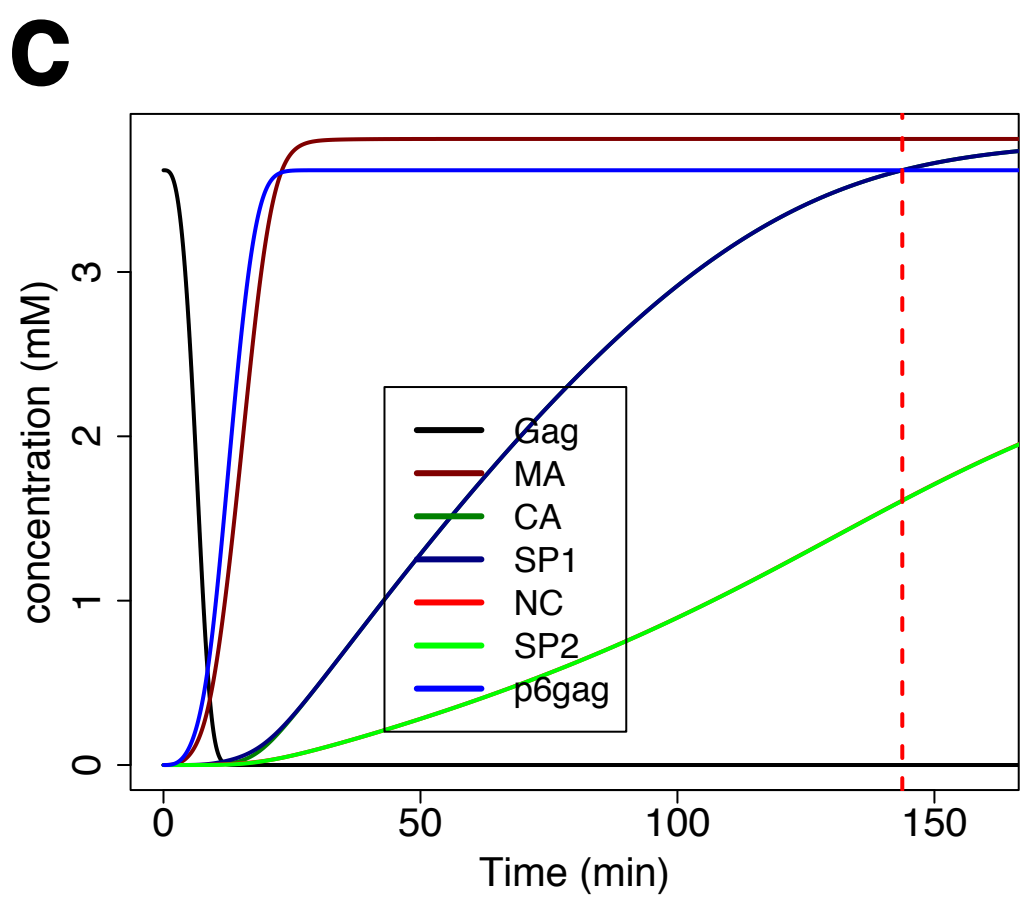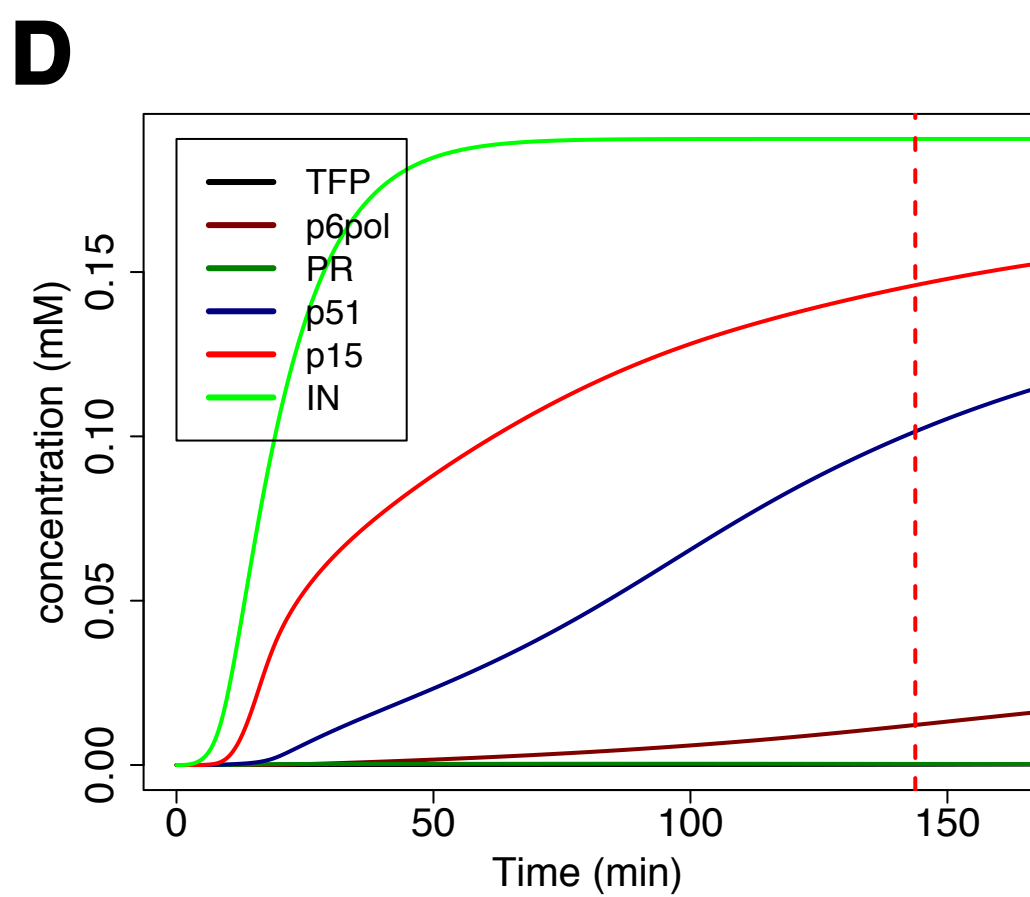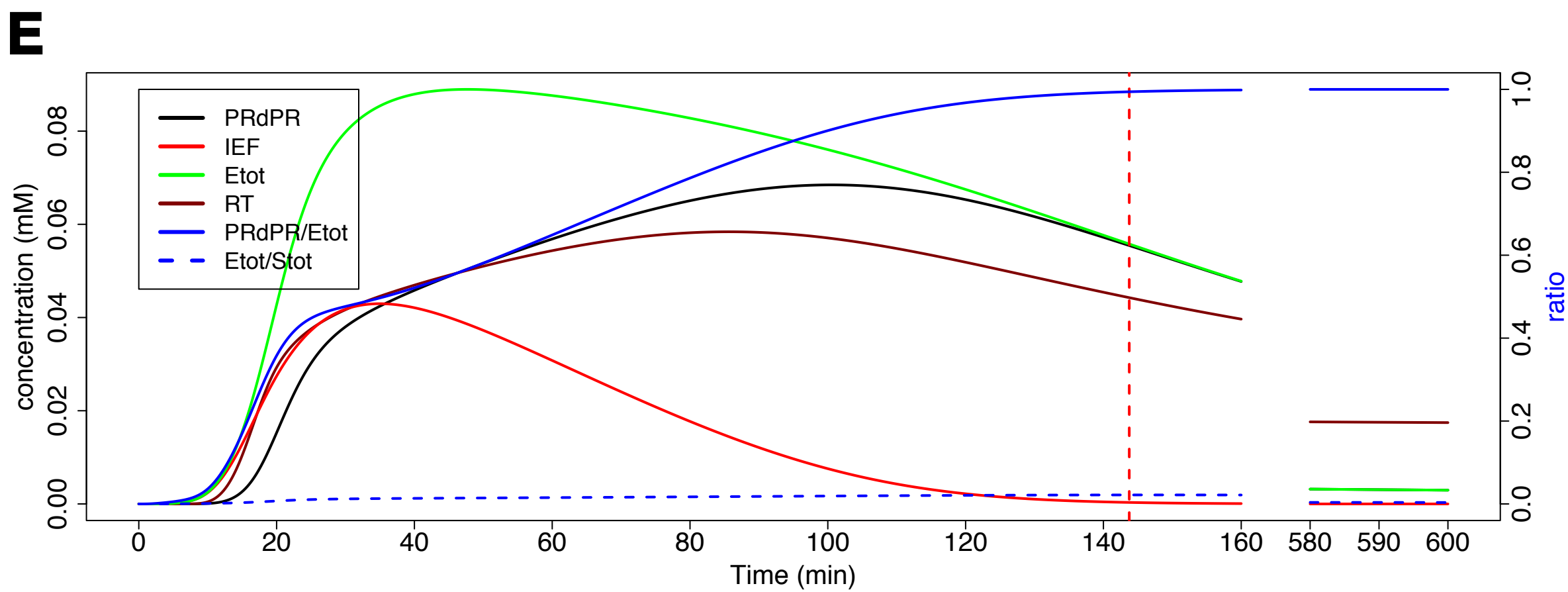

Supplement: Figure S7 — The time course of simulated Gag and Gag-Pol processing, using kinetic rate constants estimated based on full-length Gag cleavage. (A) Virus maturation time (VMT) (dashed red line in all panels) is still triggered by the decay of the CA.SP1 fragment (blue line; threshold of trans-dominant inhibition of particle maturation indicated by dashed horizontal line) and is not limited by the availability of liberated CA molecules (green line; threshold of one capsid unit corresponding to 1,500 CA molecules per particle is indicated by solid horizontal line), but occurs much later than with the default parameters. (B) Generation of catalytically active intermediate dimeric forms containing PR. (C) Decay of Gag substrate (black line) and accumulation of final Gag cleavage products. (D) Accumulation of final Pol cleavage products. (E) Enzyme concentrations and related metrics. The ratio PRdPR/Etot indicates the relative contribution of mature PR dimers to the proteolytic activity. The ratio Etot/Stot of the total concentration of active enzyme forms and the total concentration of uncleaved cleavage sites stays below one throughout the simulated time course, which justifies the use of Michaelis-Menten kinetics. Etot – total proteolytic activity; Stot – all uncleaved cleavage sites; IEF – all active intermediate enzyme (PR) forms; RT: p51/p66 heterodimer. All other dimers are indicated in the form M1dM2, where M1,2 are the monomers. Initial concentrations of Gag and Gag-Pol were set to reflect the quantities within a single virion; cleavage rates in Gag were parameterized as in [39]; all other parameters were set as in Table 1. (PDF) [file pcbi.1003103.s007.pdf]

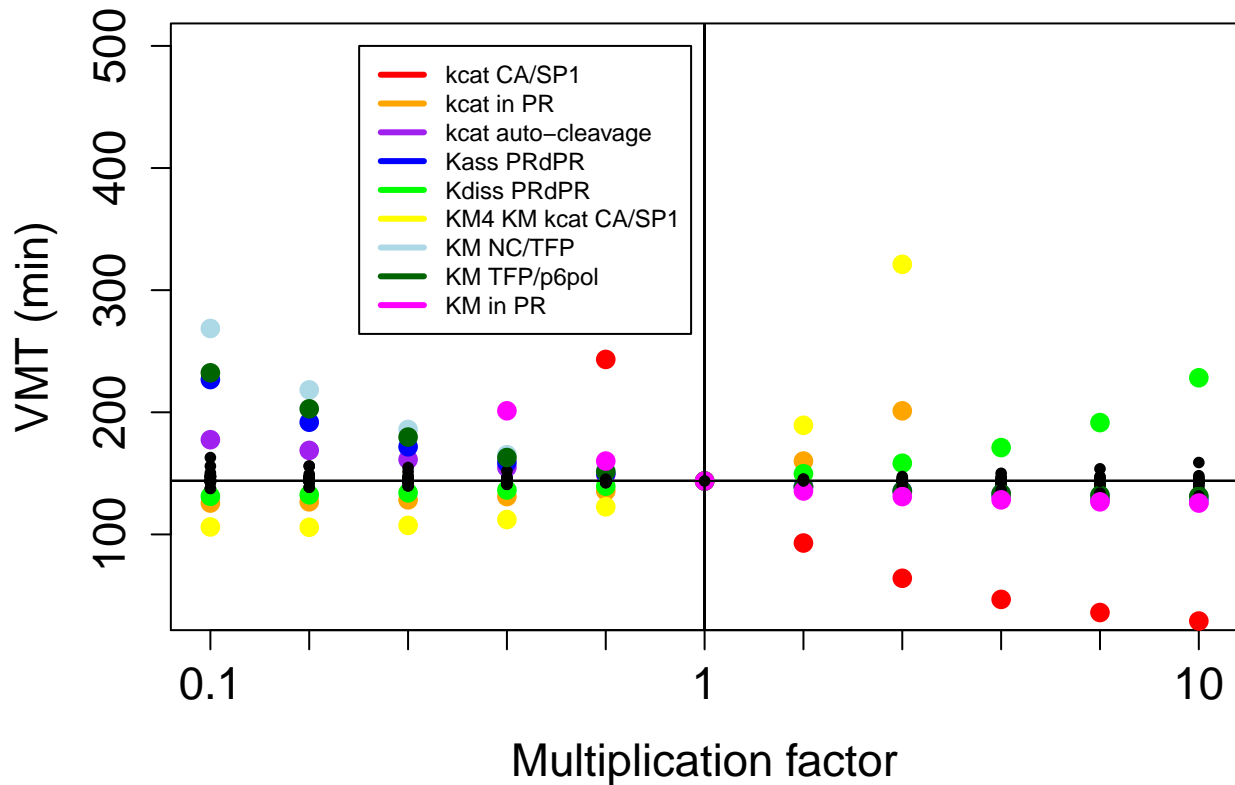

Supplement: Figure S8 — The effect of single parameter variation on VMT using an alternative set of kinetic rate constants. (PDF) [file pcbi.1003103.s008.pdf]
