# Supplementary material for: Gag-Pol Processing during HIV-1 Virion Maturation: A Systems Biology Approach
Source: PLoS Comput Biol. 2013 Jun 6;9(6):e1003103. doi: 10.1371/journal.pcbi.1003103 (PMC3675044; doi:10.1371/journal.pcbi.1003103)
Supplement: Text S1 — Estimation of catalytic rate constants. (PDF) [file pcbi.1003103.s009.pdf]

Gag-Pol Processing during HIV-1 Virion Maturation: a Systems Biology Approach

Supplement: Text S1. Estimation of catalytic rate constants

Balázs Könnyű[1], S. Kashif Sadiq[2], Tamás Turányi[3], Rita Hírmondó[1,4], Barbara Müller[5], Hans-Georg Kräusslich[5], Peter V. Coveney[6] and Viktor Müller[1,7]

[1]Institute of Biology, Eötvös Loránd University, Budapest, Hungary

[2]Computational Biophysics Laboratory (GRIB-IMIM), Universitat Pompeu Fabra, Barcelona, Spain

[3]Institute of Chemistry, Eötvös Loránd University, Budapest, Hungary

[4]Institute of Enzymology, Hungarian Academy of Sciences, Budapest, Hungary

[5]Department of Infectious Diseases, Virology, University of Heidelberg, Heidelberg, Germany

[6]Centre for Computational Science, Christopher Ingold Laboratories, University College London, London, UK

[7]Research Group of Theoretical Biology and Evolutionary Ecology, Eötvös Loránd University and the Hungarian Academy of Sciences, Budapest, Hungary

We estimated the catalytic rate constants for the NC/TFP and the TFP/p6^{pol} cleavage sites from data read from Figure 2 of [1], which presented substrate turnover over time from *in vitro* cleavage of synthetic oligopeptides containing the NC/TFP or TFP/p6^{pol} cleavage sites, respectively, catalyzed by HIV-1 PR. We used the classic framework of enzyme kinetics:

$$\frac{dE}{dt} = -k_a ES + \left(k_d + k_{cat}\right)C \tag{1}$$

$$\frac{dS}{dt} = -k_a ES + k_d C \tag{2}$$

$$\frac{dC}{dt} = k_a ES - \left(k_d + k_{cat}\right)C \tag{3}$$

$$\frac{dP}{dt} = k_{cat} C \tag{4}$$

where E, S, C and P denote the concentrations of free enzyme (in this case: PR dimers), free substrate, enzyme–substrate complexes and cleaved products, respectively. The parameters k_a and k_d denote the rate constants of association and dissociation of the complex, and k_{cat} denotes the rate constant of cleavage of the complex. It holds that $S+C+P=S_{tot}$ and $E+C=E_{tot}$ throughout the reaction. Initial conditions in both experiments were $S_0=S_{tot}$=0.14 mM (substrate concentration) and $E_0=E_{tot}$= 2.25mM (concentration of PR dimers). The excess of enzyme over substrate implies that the QSS approximation for the enzyme–substrate complex is not likely to hold: $E_{tot}/(S_{tot}+K_M)$ is likely greater than one, considering that the K_M constants for the cleavage sites with available empirical estimates have a median of 0.05 mM and a maximum of 1.2 mM. However, the slow cleavage of both cleavage sites suggests that the catalytic rate constant of cleavage is likely to be smaller than the rate constant of dissociation of the enzyme–substrate complex ($k_{cat}<k_d$), which allows us to use a quasi-steady-state approximation for the concentration of free substrate (as in the original equilibrium approximation of Michaelis and Menten [2]). Using this approximation (dS/dt = 0) and the further approximation of $E \approx E_{tot}$ (based on $E_{tot} >> S_{tot} \geq C$), we obtain that the decay of total (free and complexed) uncleaved substrate ($S^*=S+C$) follows a simple exponential decay with rate $\lambda \approx k_{cat}*E_{tot}/(E_{tot}+K_D)$, where $K_D = k_d/k_a \approx K_M = (k_d+k_{cat})/k_a$, if $k_{cat}<<k_d$. The data in [1] show the concentration of cleaved products (P) over time, from which we calculated $S^*=S_{tot}$ - P, and then estimated the exponential rate, λ, by fitting exponential decay to the points (Figure S9A,D). From λ, k_{cat} can be calculated for any fixed value of K_M, but it is not possible to obtain independent estimates for both parameters. We verified that the approximated model can be fitted to the experimental data in a broad range of K_M values (Figure S9B,E), and then fixed K_M at the median of the values for cleavage sites with empirical estimates (0.05 mM), to obtain for k_{cat} 1.9×10^{-5} and 2.0×10^{-4} s^{-1} for the NC/TFP and the TFP/p6^{pol} cleavage sites, respectively. We then used the full model (Equations 1-4) to test whether the approximations used in the estimation were valid. For any pair of corresponding K_M and k_{cat} values, one of k_a or k_d can be calculated when the other parameter is known. We first fixed k_d = 1 s^{-1} (as a

rough consensus of estimates obtained for different substrates and with different methods in [3] and [4]) and calculated $k_a = (k_{cat}+k_d)/K_M$ for all pairs of K_M and k_{cat} values. When parameterized with these estimates, the full model yields a very good fit to the experimental data (Figure S9C,F). Fixing k_a and calculating k_d yielded equally good fits. We also verified that the criterion for the quasi-steady-state approximation, $k_{cat} << k_d$, was indeed valid in all cases tested. Finally, we repeated the estimation using rounded time points (whole hours/minutes, to correspond to probable original measurement times) instead of the fractional values that were read directly from the figure in [1]: the estimates for the catalytic rate constants changed very little (<5%). We thus conclude that our estimation procedure is robust. Note also that the effect of the kinetics of both cleavage sites is largely confined to the TFP and p6^{pol} fragments, and its influence on virion maturation is negligible.

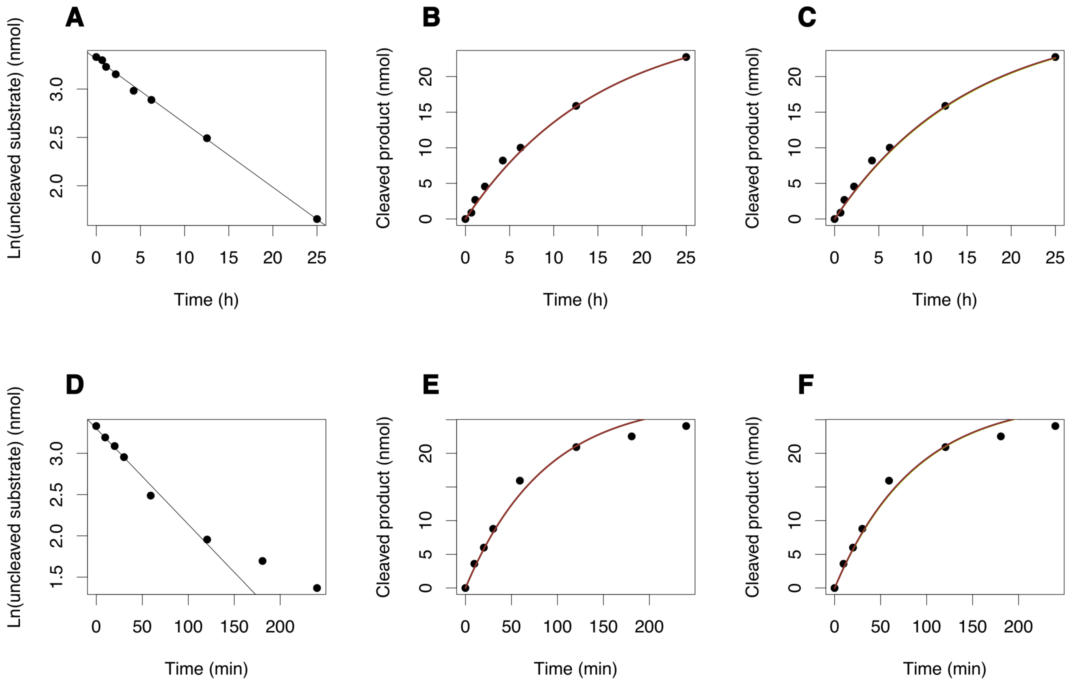

Figure S9. **Estimation of kinetic rate constants for the NC/TFP (top row) and the TFP/p6^{pol} (bottom row) cleavage sites.** Full dots represent data points read from Figure 2 of [1]. Panels A and D show exponential decay fitted to the concentration of uncleaved substrate: for the NC/TFP cleavage site (A) the regression on log transformed data had $R^2 = 1.00$ and $p=2\times10^{-9}$; for the TFP/p6^{pol} cleavage site (B), we omitted the last two data points that

failed to fit to the expected exponential decay, and obtained R^2=0.98, p=8×10^{-5} in the regression. Panels B and E show model fits to the data points of cleaved product, obtained assuming quasi-steady-state dynamics for free substrate; Panels C and F show fits obtained with the full kinetics model (Equations 1-4). Each of panels B,C,E,F show four graphs obtained with K_M equal to 0.05, 0.5, 5 and 50 mM/s, respectively, and the corresponding calculated values of k_{cat} (1.88, 2.25, 5.94, 42.77 ×10^{-5} for NC/TFP; 1.98, 2,36, 6.23, 44.92 ×10^{-4} for TFP/p6^{pol}): the graphs are completely overlaid. In Panels C,F, k_d = 1 s^{-1} was fixed and k_a was calculated as $k_a = (k_{cat}+k_d)/K_M$ for all pairs of K_M and k_{cat} values.

Supplementary references

1. Ludwig C, Leiherer A, Wagner R (2008) Importance of protease cleavage sites within and flanking human immunodeficiency virus type 1 transframe protein p6* for spatiotemporal regulation of protease activation. J Virol 82: 4573-4584.
2. Michaelis L, Menten ML (1913) Die Kinetik der Invertinwirkung. Biochem Z 49: 333–369.
3. Furfine ES, D'Souza E, Ingold KJ, Leban JJ, Spector T, et al. (1992) Two-step binding mechanism for HIV protease inhibitors. Biochemistry 31: 7886-7891.
4. Katoh E, Louis JM, Yamazaki T, Gronenborn AM, Torchia DA, et al. (2003) A solution NMR study of the binding kinetics and the internal dynamics of an HIV-1 protease-substrate complex. Protein Sci 12: 1376-1385.
5. Pettit SC, Henderson GJ, Schiffer CA, Swanstrom R (2002) Replacement of the P1 amino acid of human immunodeficiency virus type 1 Gag processing sites can inhibit or enhance the rate of cleavage by the viral protease. J Virol 76: 10226-10233.
6. Sadiq SK, Könnyü B, Müller V, Coveney PV (2011) Reaction kinetics of catalyzed competitive heteropolymer cleavage. J Phys Chem B 115: 11017-11027.
